# Supplementary material for: The Chinese mitten crab genome provides insights into adaptive plasticity and developmental regulation
Source: Nat Commun. 2021 Apr 22;12:2395. doi: 10.1038/s41467-021-22604-3 (PMC8062507; doi:10.1038/s41467-021-22604-3)
Supplement: Supplementary file 1 — Supplementary Information [file 41467_2021_22604_MOESM1_ESM.pdf]

Supplementary Information

The Chinese mitten crab genome provides insights into adaptive plasticity and developmental regulation

Supplementary Figures

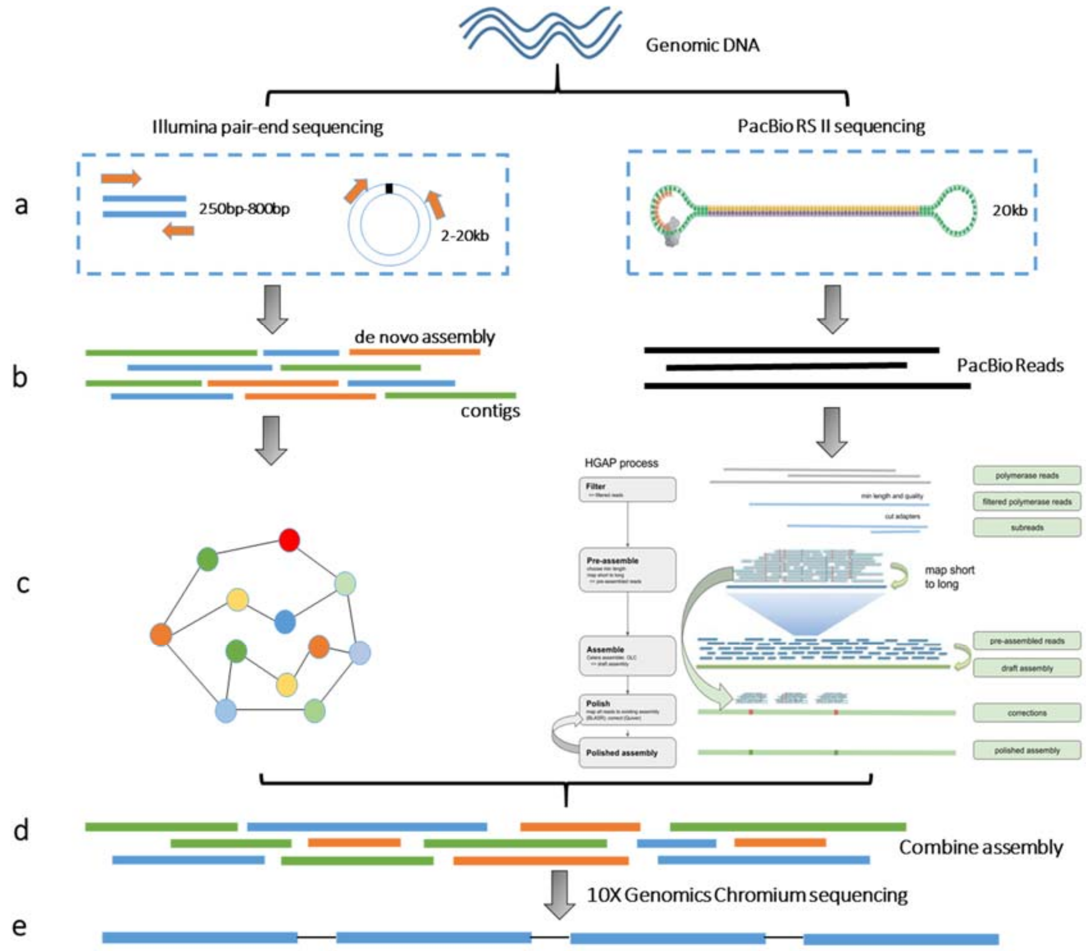

Supplementary Figure 1. Strategy for *E. sinensis* genome assembly. (a) Illumina short and long-insert libraries were constructed, and ~258× coverage of sequencing reads were generated. PacBio RS II sequencing was performed on SMRT cells to generate ~55 Gb PacBio long reads with ~35× coverage. (b) The Illumina data were

assembled into contigs using Discovar denovo with optimizing parameters. (c) Graph module account *k*-mer frequency from Illumina reads, with the unique *k*-mer extracted for alignment and the removal of duplication in the latter modules. The PacBio data were assembled by Falcon. (d) Combination of the Illumina and PacBio contigs by HABOT2. (e) Scaffolds were built using ~106× coverage of 10x Genomics Chromium data.

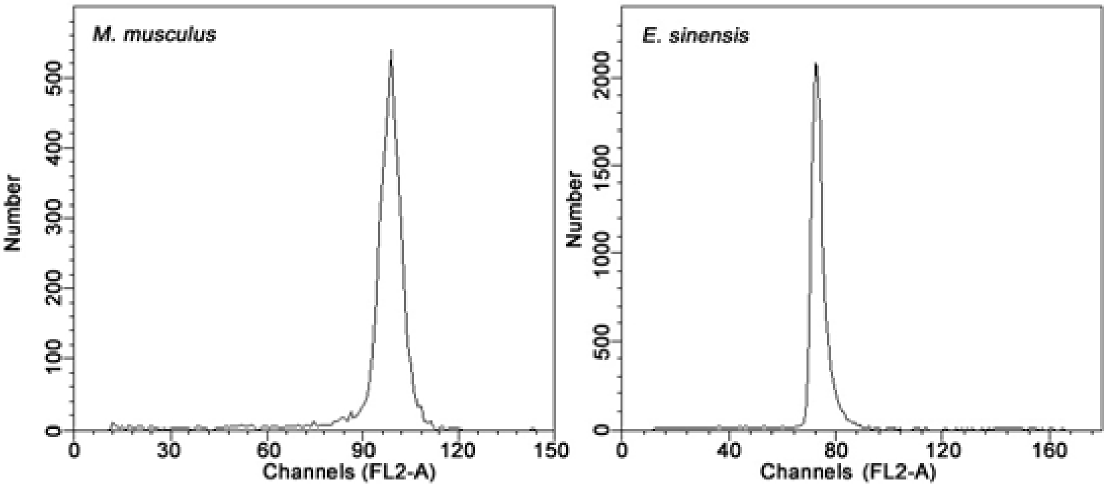

Supplementary Figure 2. Genome size estimation of *E. sinensis* by flow cytometric analysis. The results provided an estimation of 1.81 pg/1C (peak at 73.11), which was equivalent to 1.77 Gb per haploid genome of *E. sinensis* based on the formula: 1 pg = 0.978 Gb. The blood of *Mus musculus* (2.45 pg/1C, peak at 98.76) was used as an internal standard.

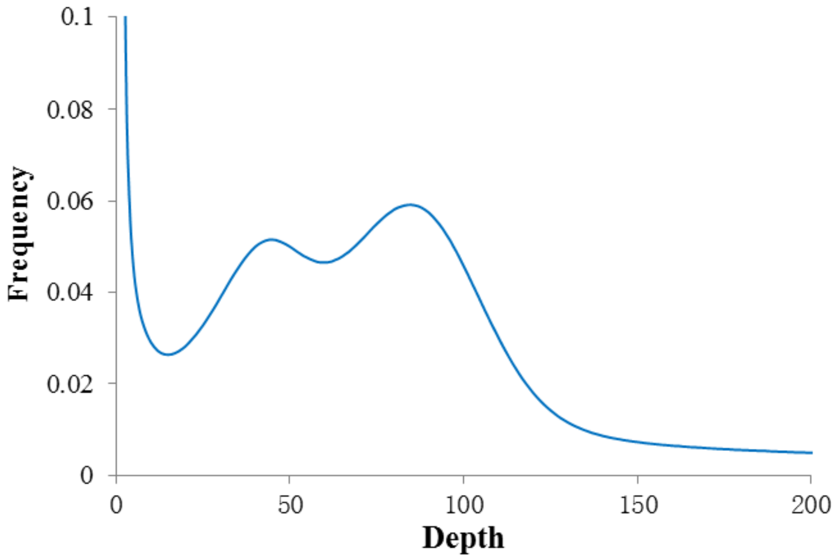

Supplementary Figure 3. Distribution of 21-mer frequency in the *E. sinensis* genome. The paired-end reads were derived from the sequencing of DNA libraries, with insert sizes of 250 bp and 500 bp. Two peaks rather than one were observed, indicating high genomic heterozygosity. The genome size of *E. sinensis* was estimated as: (total number of 21-mers)/(homozygous peak depth) = 1.45 Gb.

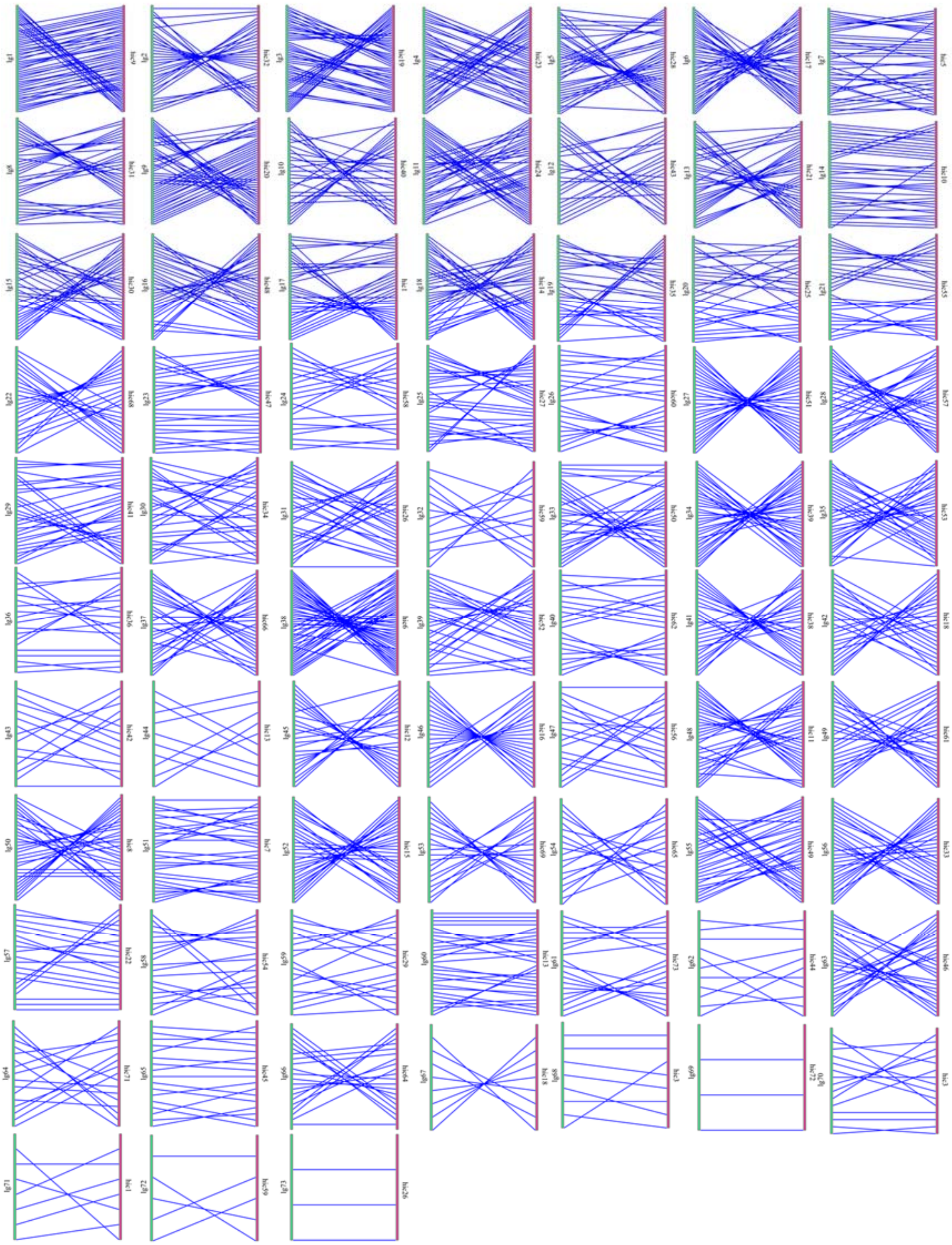

Supplementary Figure 4. 67 chromosomes assembled using linkage map were consistent with 73 chromosomes assembled using Hi-C data.

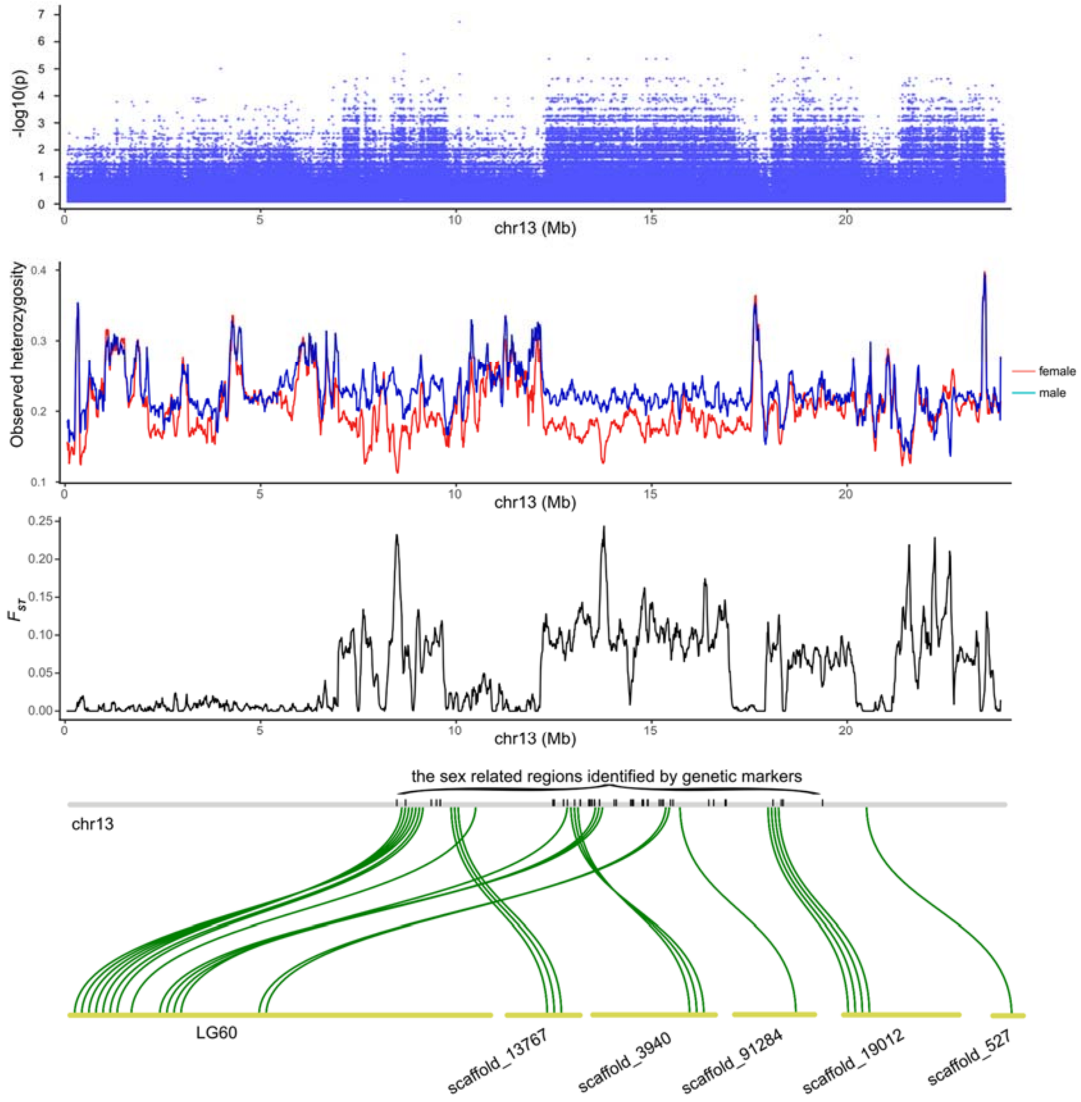

Supplementary Figure 5. The sex-related genes in chr13, and three parameters (F_{ST}, H_{obs}, and -log10(p-value)) along chromosome chr13. a) In the first graph below, the green lines represent the 26 sex-related genes in chr13. The sex-related makers previously identified by linkage map are drawn in black whiskers. b) The second graph below shows the F_{ST} along chromosome chr13. c) The third graph below shows the observed heterozygosity (H_{obs}) of female and male populations along chromosome chr13. d) The negative logarithm of GWAS p-value along chromosome chr13.

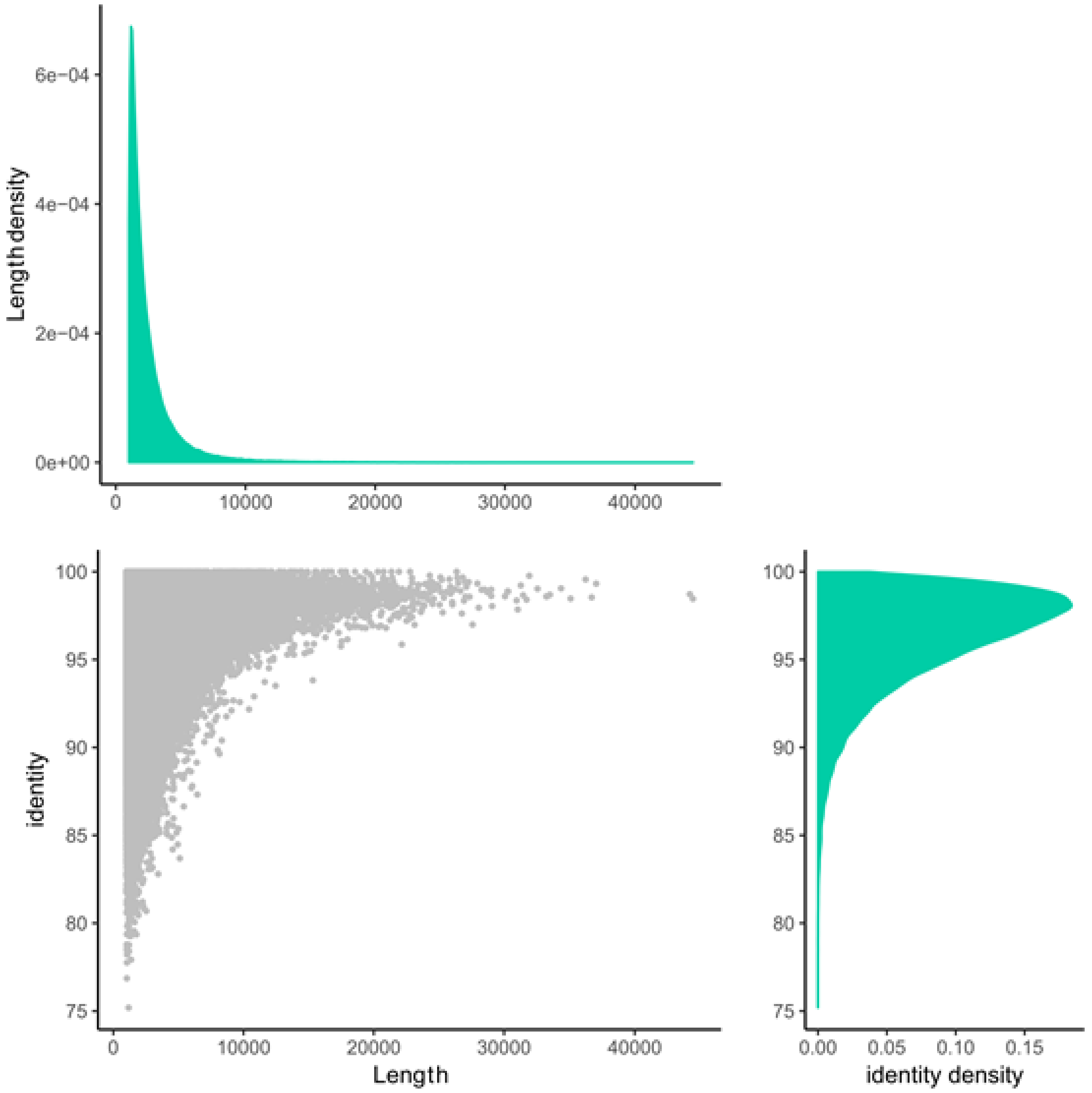

Supplementary Figure 6. Statistics of the alignment using NUCmer between the male genome in our study and the female genome in a previous study. The middle scatter plot represents the length and identity of each aligned segment (>1kb). The graph on the right shows the frequency distribution of the alignment identity, and the distribution of the alignment length is shown at the top.

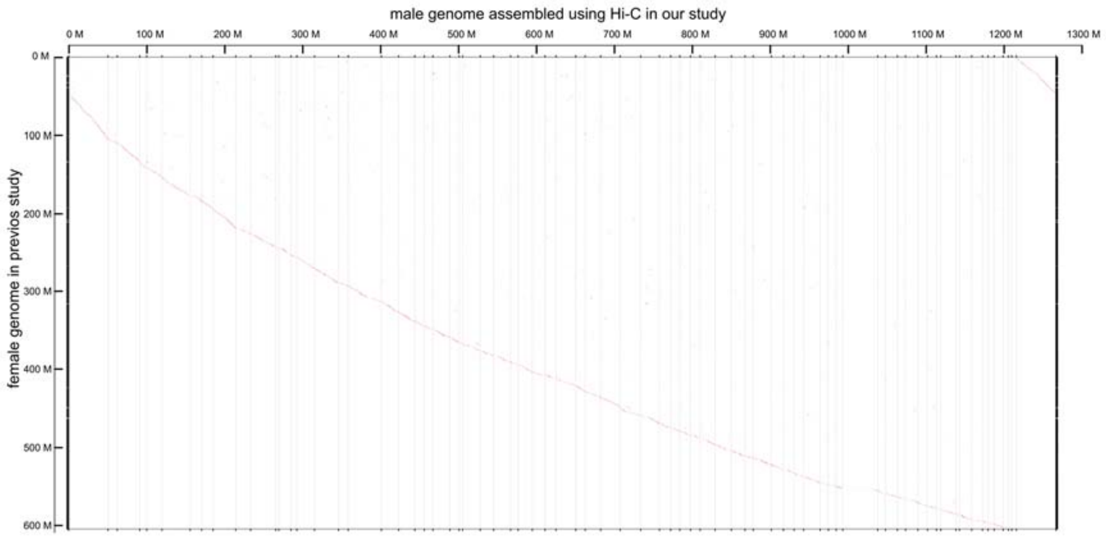

Supplementary Figure 7. The synteny of the male genome in our study and the female genome in a previous study. The alignment was re-ordered to the diagonal for ease of viewing. Only the alignment fragments with lengths more than 5kb are shown. The forward matches are displayed in red and the reverse matches in blue. x-axis: our male genome, y-axis: female genome in a previous study.

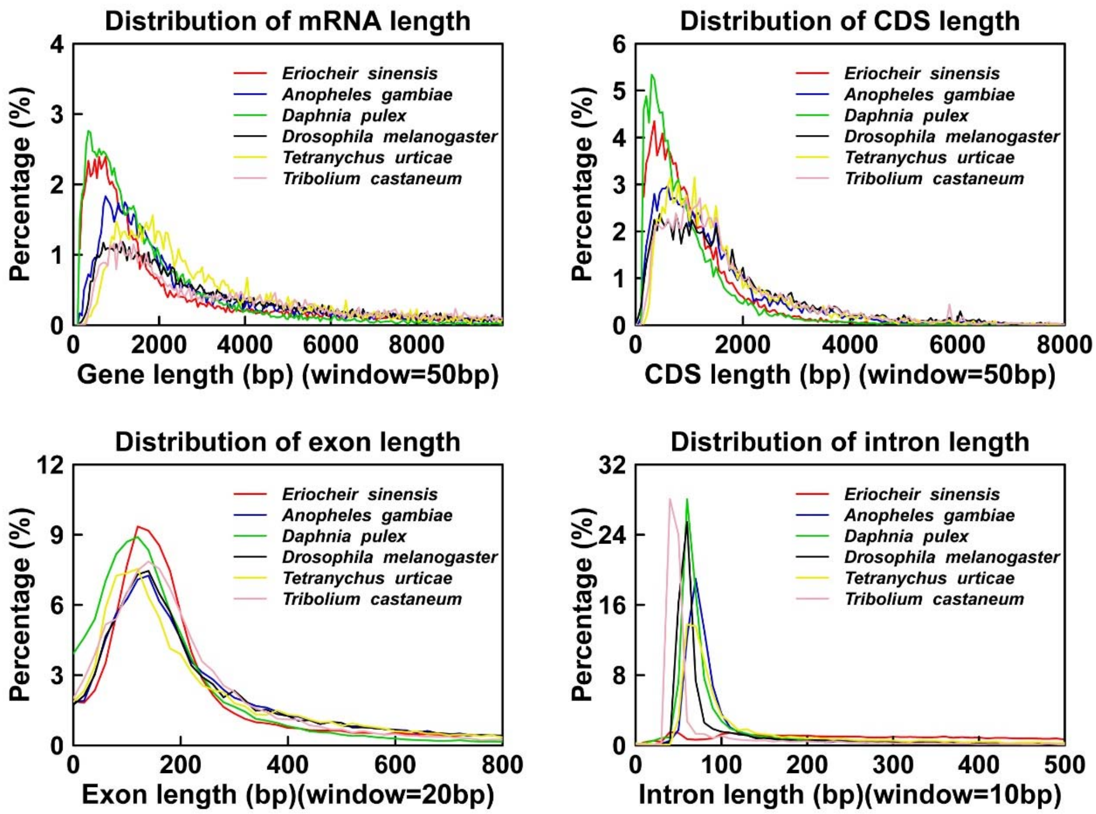

Supplementary Figure 8. Characterization of different genic regions in *E. sinensis* and five other arthropods.

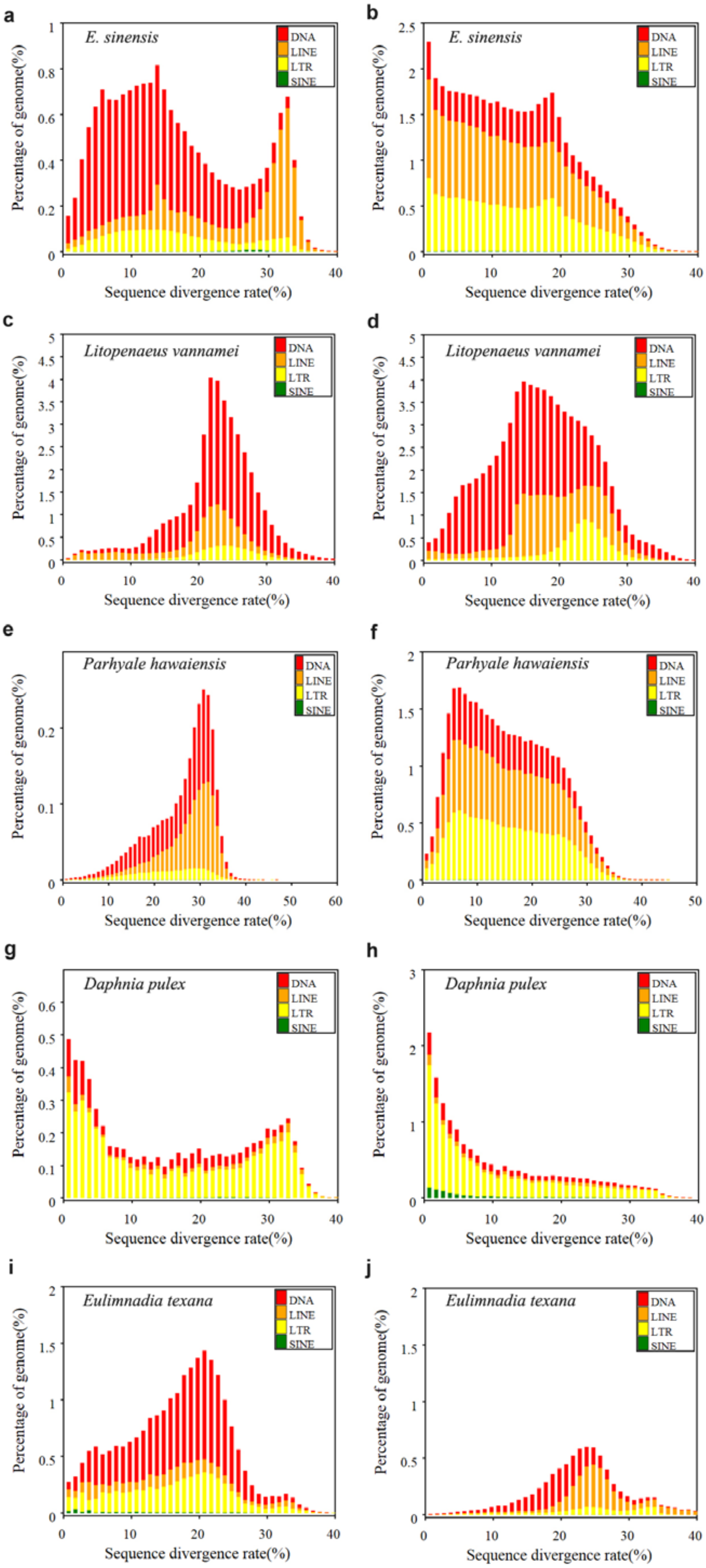
a
E. sinensis
b
E. sinensis
c
Litopenaeus vannamei
d
Litopenaeus vannamei
e
Parhyale hawaiensis
f
Parhyale hawaiensis
g
Daphnia pulex
h
Daphnia pulex
i
Eulimnadia texana
j
Eulimnadia texana
DNA
LINE
LTR
SINE
Percentage of genome(%)
Sequence divergence rate(%)

Supplementary Figure 9. Divergence rates of transposable elements (TEs) identified by homology (a, c, e, g and i) or *de novo* prediction (b, d, f, h and j). *E. sinensis* (a, b) shows a higher proportion of active TEs (divergence rate < 10%) than those of the crustaceans *Litopenaeus vannamei* (c, d), *Parhyale hawaiensis* (e, f), *Daphnia pulex* (g, h) and *Eulimnadia texana* (i and j).

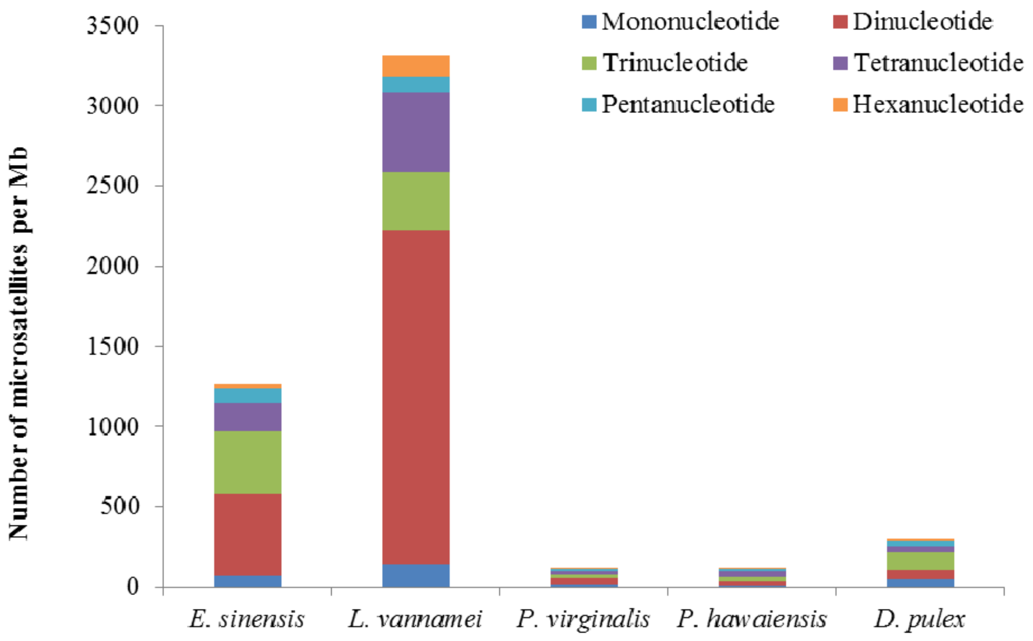

Supplementary Figure 10. Comparison of full-genome microsatellite density between *E. sinensis* and four other crustaceans.

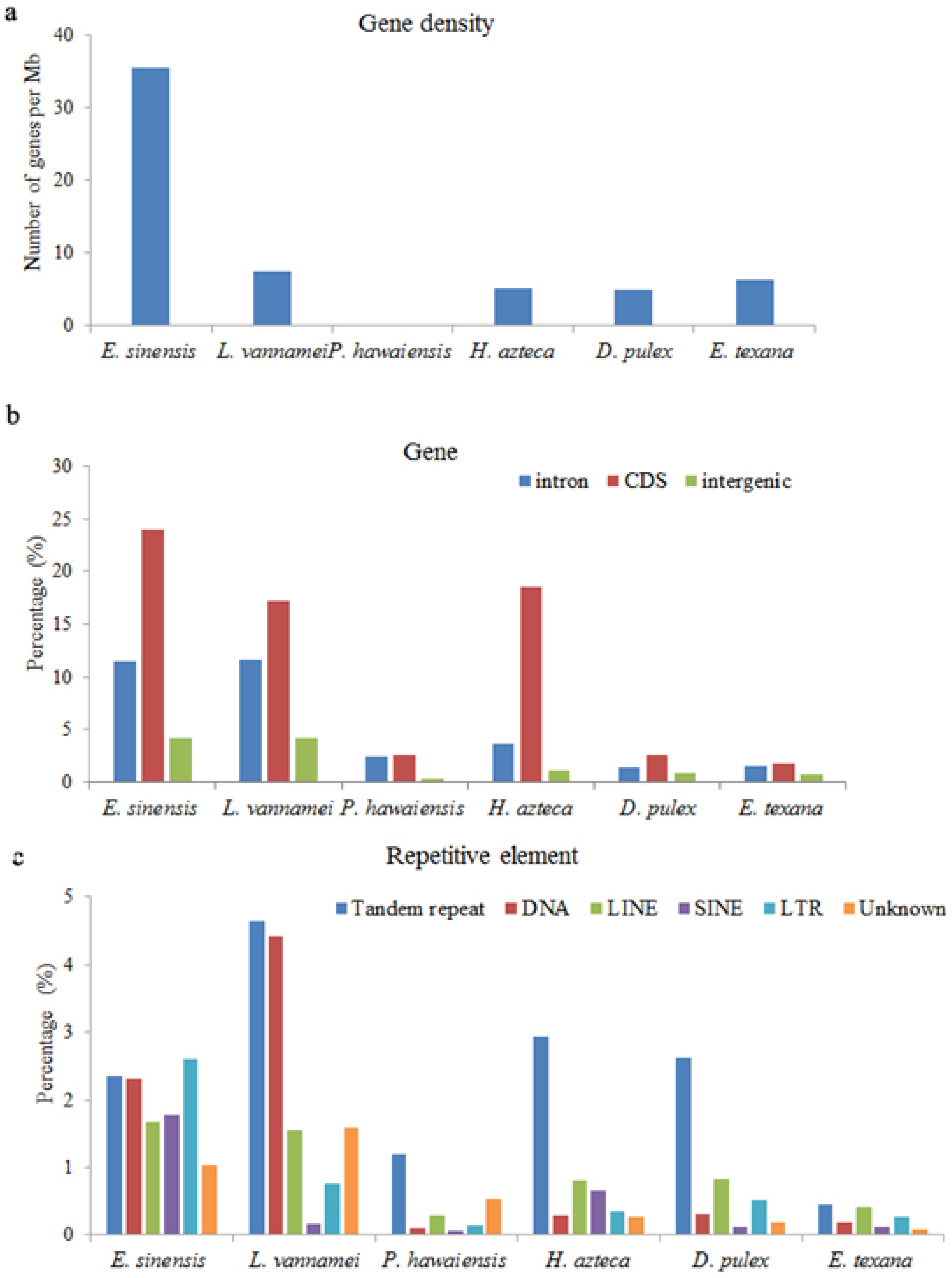

Supplementary Figure 11. Gene density (a) and percentage of GC-rich regions (> 60%) in respective classification of genes (b) and repetitive elements (c) in the genomes of six representative crustacean species.

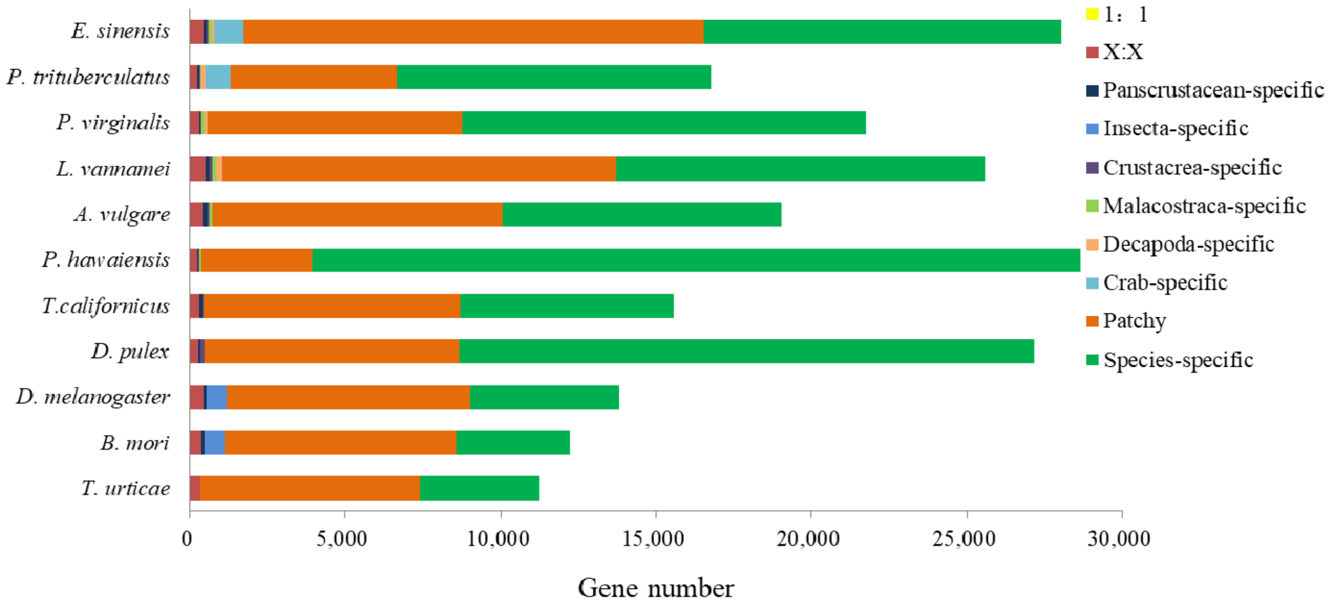

Supplementary Figure 12. Comparison of the gene repertoire of 11 arthropod genomes. Bars are subdivided to represent different types of orthology relationships. “1:1” indicates single-copy genes; “X:X” indicates orthologous genes present in multiple copies in all the 11 species, where X denotes one or more orthologs per species; and “Patchy” indicates the existence of other orthologs that are present in at least one genome.

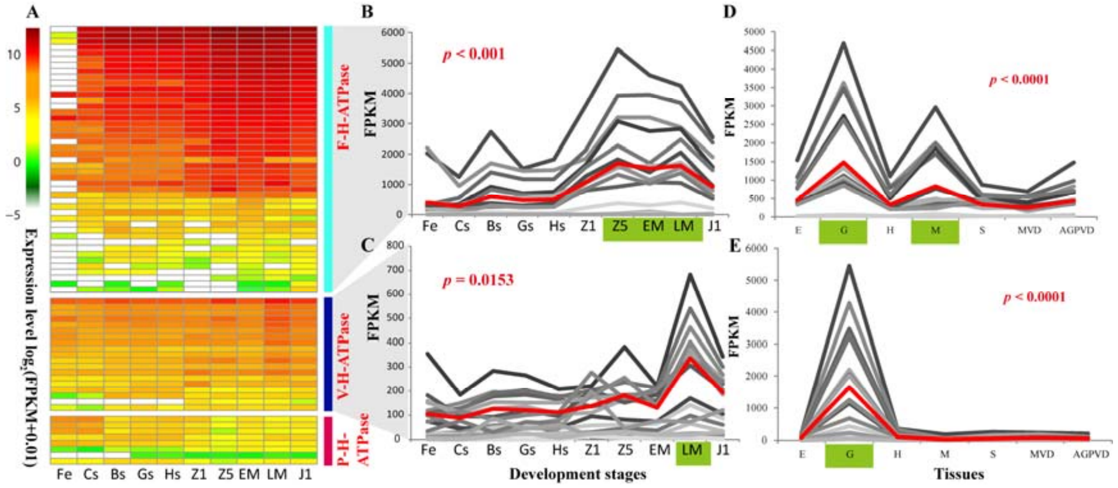

Supplementary Figure 13. The expression patterns of three types of H^+ pump. (A) Heatmap displayed the expression patterns of three types of H^+ pump during the following life history stages: fertilized egg stage (Fe), cell stage (Cs), blastula stage (Bs), gastrula stage (Gs), heartbeat stage (Hs), first zoeal stage (Z1), fifth zoeal stage (Z5), megalopa before desalination (EM), megalopa after desalination (LM), and first juvenile instar (J1). The megalopae underwent a process of desalination during development. The expression patterns of F-ATPases and V-ATPases during different development stages and among different tissues are displayed in four line chart plots (B-E). The gray lines indicate the expression pattern of each gene, and the red lines indicate the mean expression level. Tissues include eyestalk (E), gill (G), hepatopancreas (H), muscle (M), sexual gland (S), ED eja culatory duct (MVD) and androgenic gland with ejaculatory duct (AGPVD). The *p* value indicates the consistence of expression pattern, which was calculated using two-sided F test. A lower *p* value indicates a higher similarity of the expression pattern.

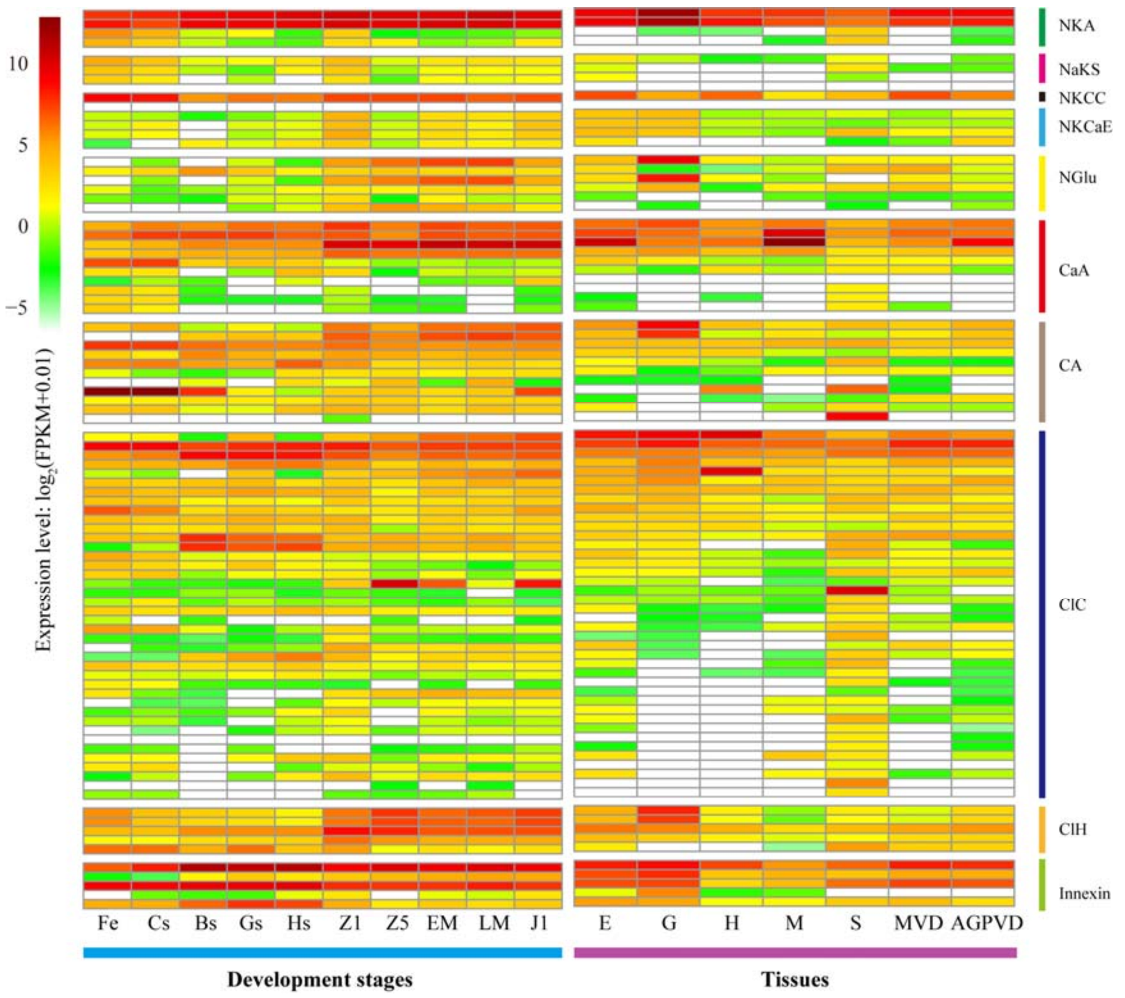

Supplementary Figure 14. Heatmap of the expression patterns of osmoregulation related genes during different life history stages and among different tissues. The osmoregulation related genes include Na^+/K^+ ATPase (NKA), Na+/K+ symporter (NaKs), $Na^+/K^+/2Cl^-$ cotransporter (NKCC), $Na^+/K^+/Ca^{2+}$ exchanger (NKCaE), sodium/glucose cotransporter (NGlu), Ca^{2+} transporting ATPase (CaA), carbonic anhydrase (CA), chloride channel protein (ClC), Na^+-independent Cl/HCO_3 exchanger (ClH), and innexin. The various life history stages were: fertilized egg stage (Fe), cell stage (Cs), blastula stage (Bs), gastrula stage (Gs), heartbeat stage (Hs), first zoeal stage (Z1), fifth zoeal stage (Z5), megalopa stage before desalination (EM), megalopa stage

after desalination (LM), and first juvenile instar (J1). The megalopae underwent a process of desalination during development. Different tissues: eyestalk (E), gill (G), hepatopancreas (H), muscle (M), sexual gland (S), ED ejaculatory duct (MVD), and androgenic gland with ejaculatory duct (AGPVD).

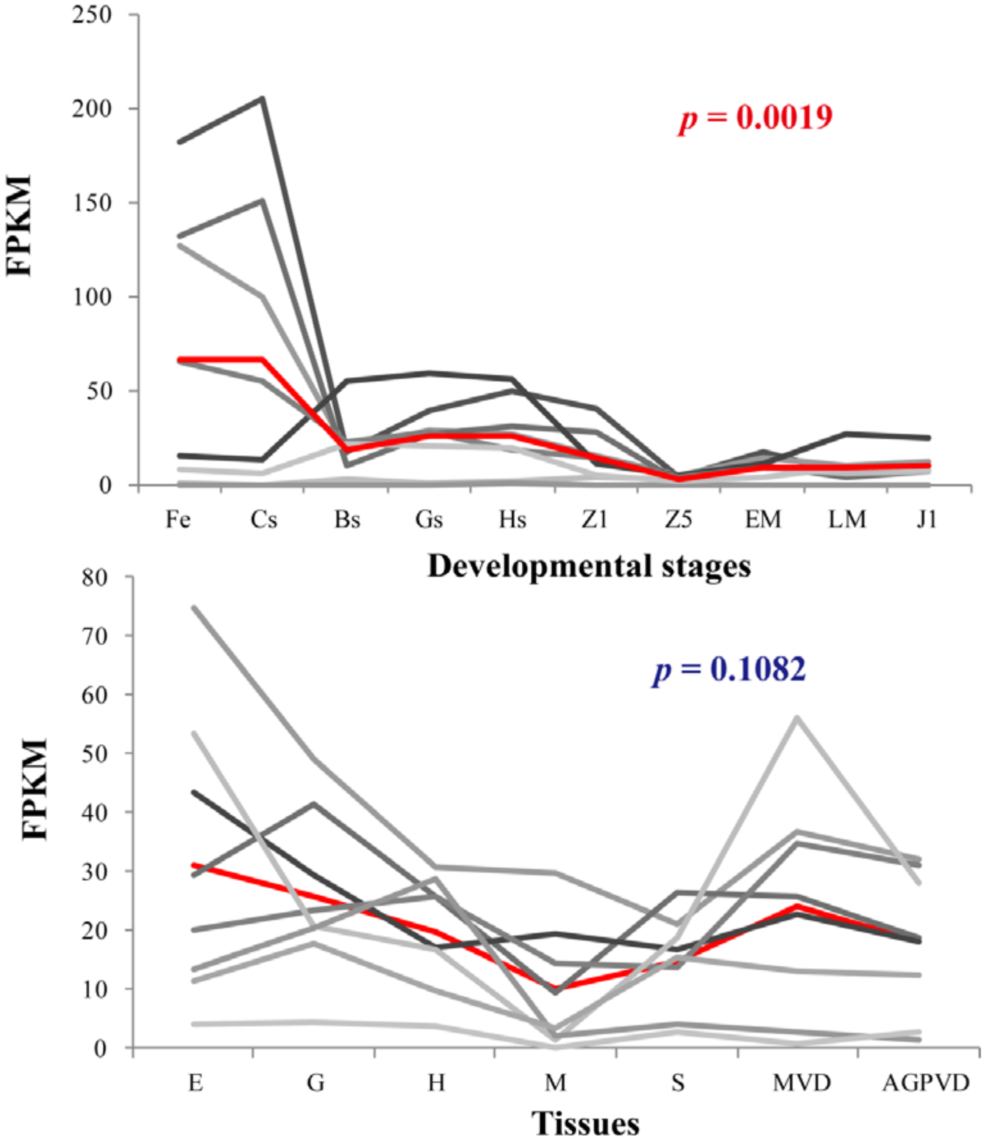

Supplementary Figure 15. The expression patterns of P-ATPases during different life history stages and among different tissues (see legend of Supplementary

Figure 14). The *p* value indicates the consistence of expression pattern, which was calculated using two-sided F test. A lower *p* value indicates a higher similarity of the expression pattern.

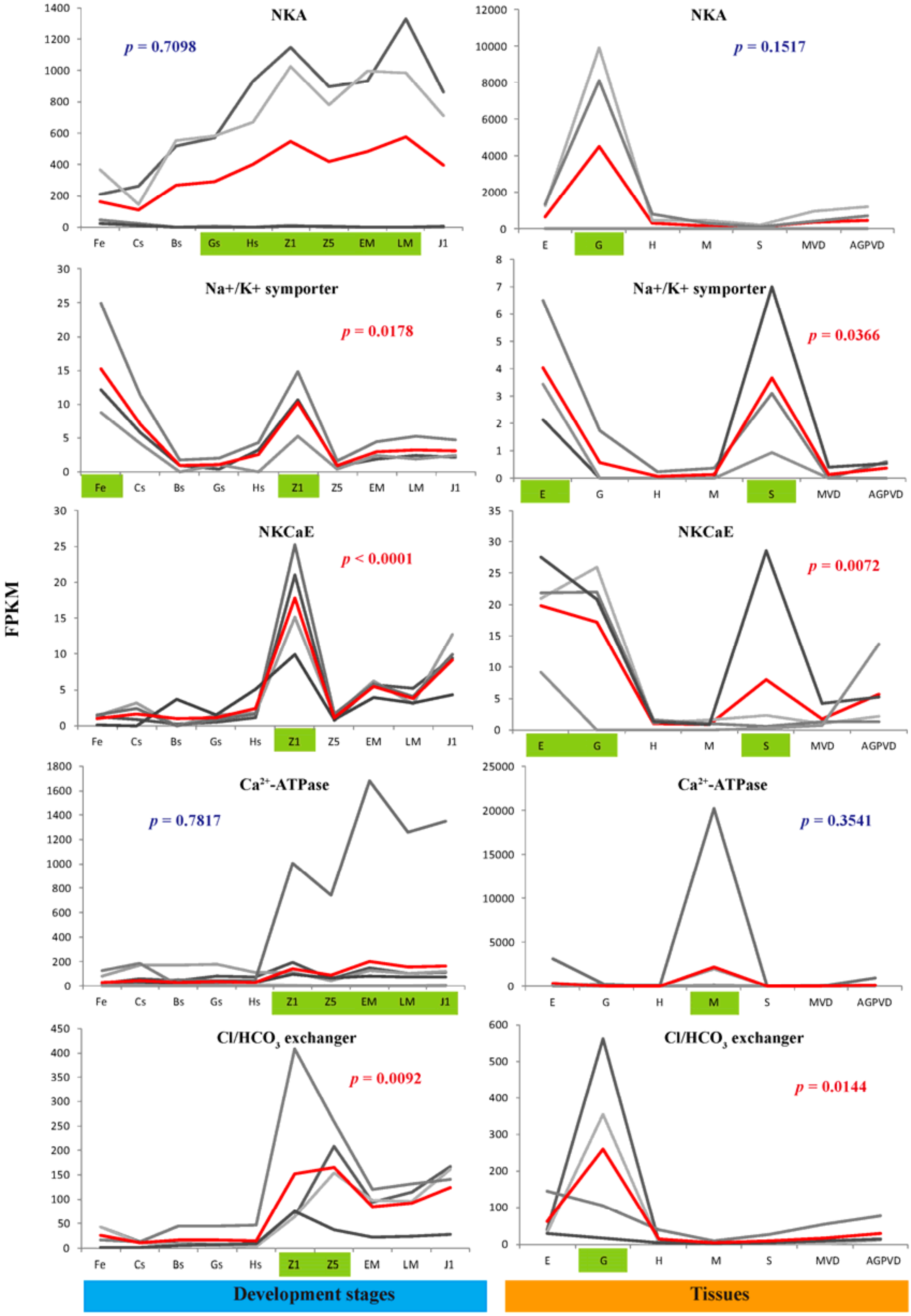

Supplementary Figure 16. The expression patterns of osmoregulation related genes during different life history stages and among different tissues (see legend of

Supplementary Figure 14). The *p* value indicates the consistence of expression pattern, which was calculated using two-sided F test. A lower *p* value indicates a higher similarity of the expression pattern.

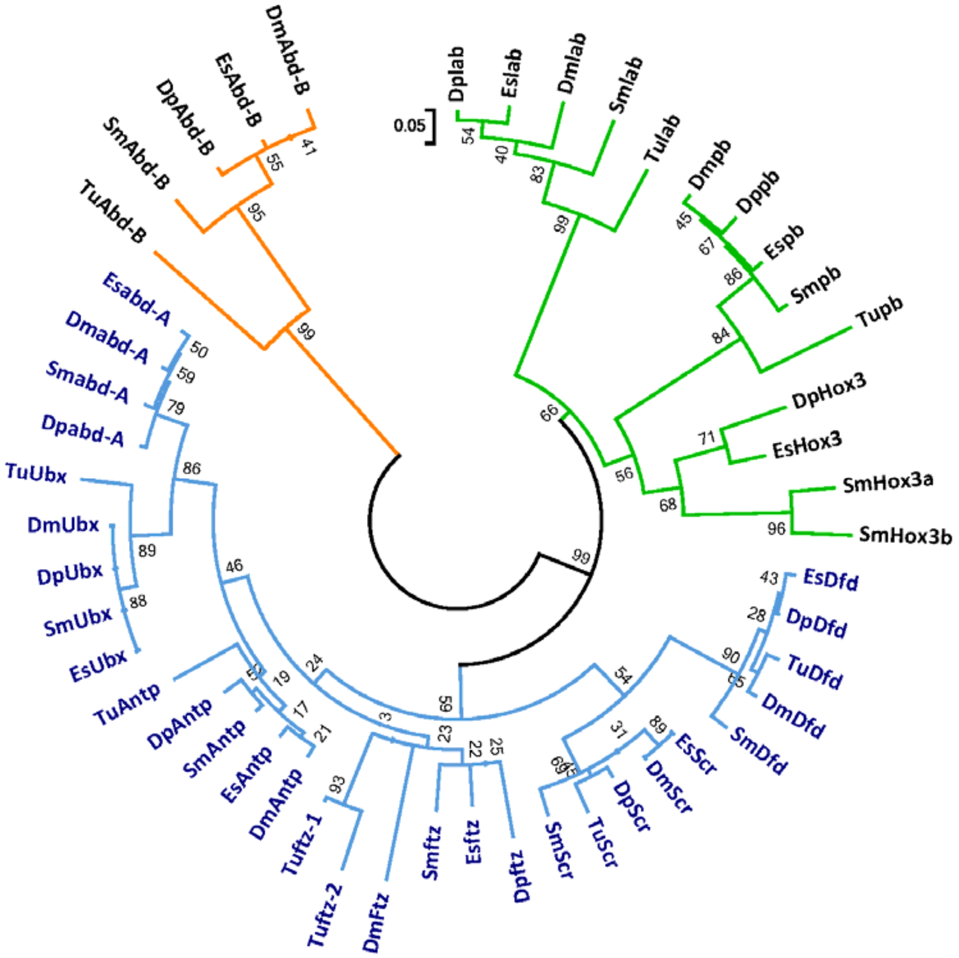

Supplementary Figure 17. Phylogenetic relationships (in protein sequences) of Hox genes from five arthropods inferred with the maximum likelihood (ML) and neighbor-joining (NJ) methods. Branch lengths and topologies were derived from ML analysis. Numbers in each branch indicate ML/NJ bootstrap values above 50%. Dm, *Drosophila melanogaster*; Dp, *Daphnia pulex*; Sm, *Strigamia maritima*; Es, *E. sinensis*; Tu, *Tetranychus urticae*.

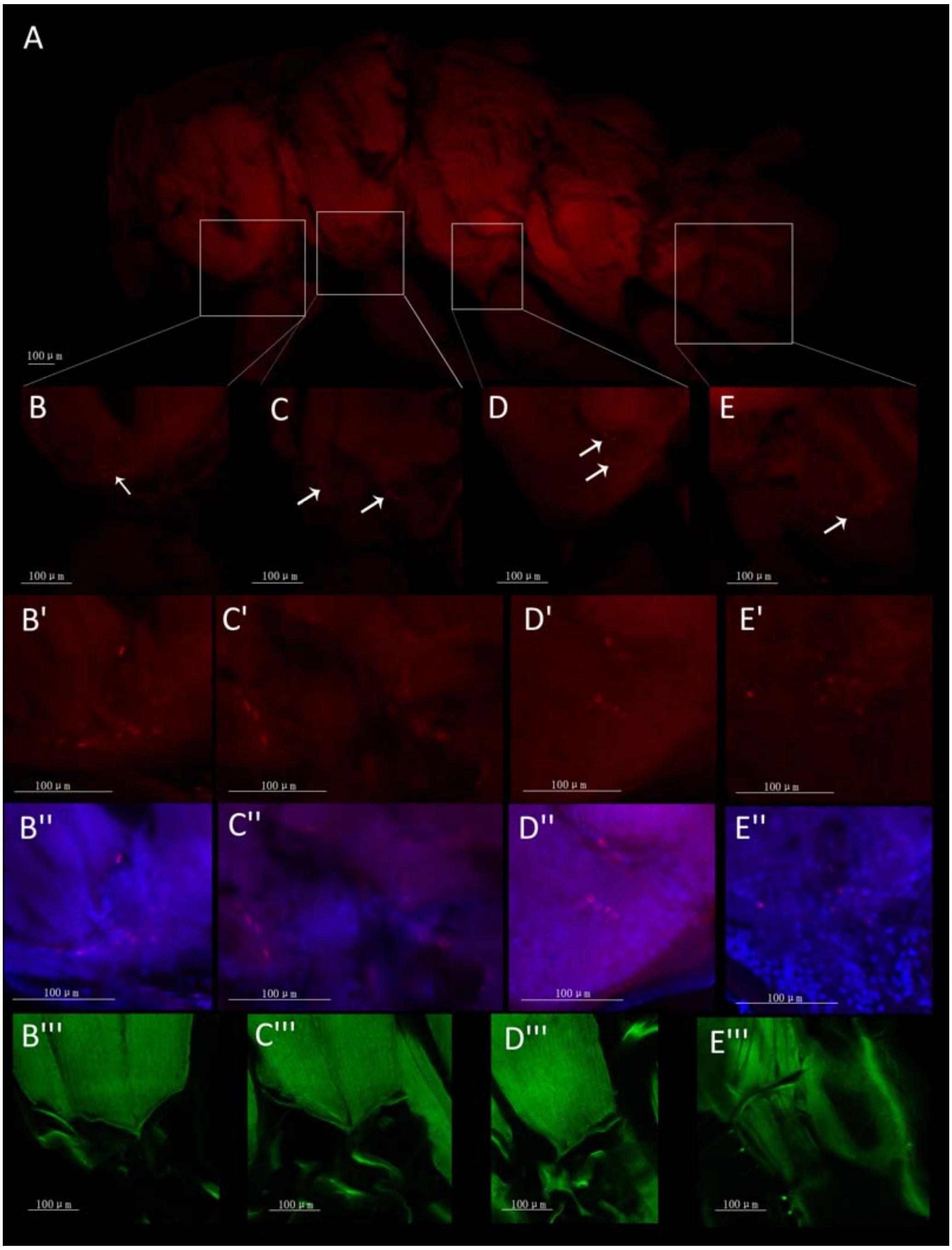

Supplementary Figure 18. ***Abd-A*** **localization in the abdomen of LM larva of** ***E. sinensis*****.** The *Abd-A* mRNA primarily located in the second, third, fourth ventral segment and the caudal segment (arrows). A. Abdomen of LM larva; B. The second ventral segment of LM larva; C. The third ventral segment of LM larva; D. The fourth ventral segment of LM larva; E. The caudal segment of LM larva; B'-E'. Figure B-E

amplification; B''-E''. Merge for 5-FAM and DAPI stained picture; B'''-E'''. Control group with GFP mRNA probe; arrows indicate positive position; scale table shows 100 μM. This experiment was repeated three times independently with similar results.

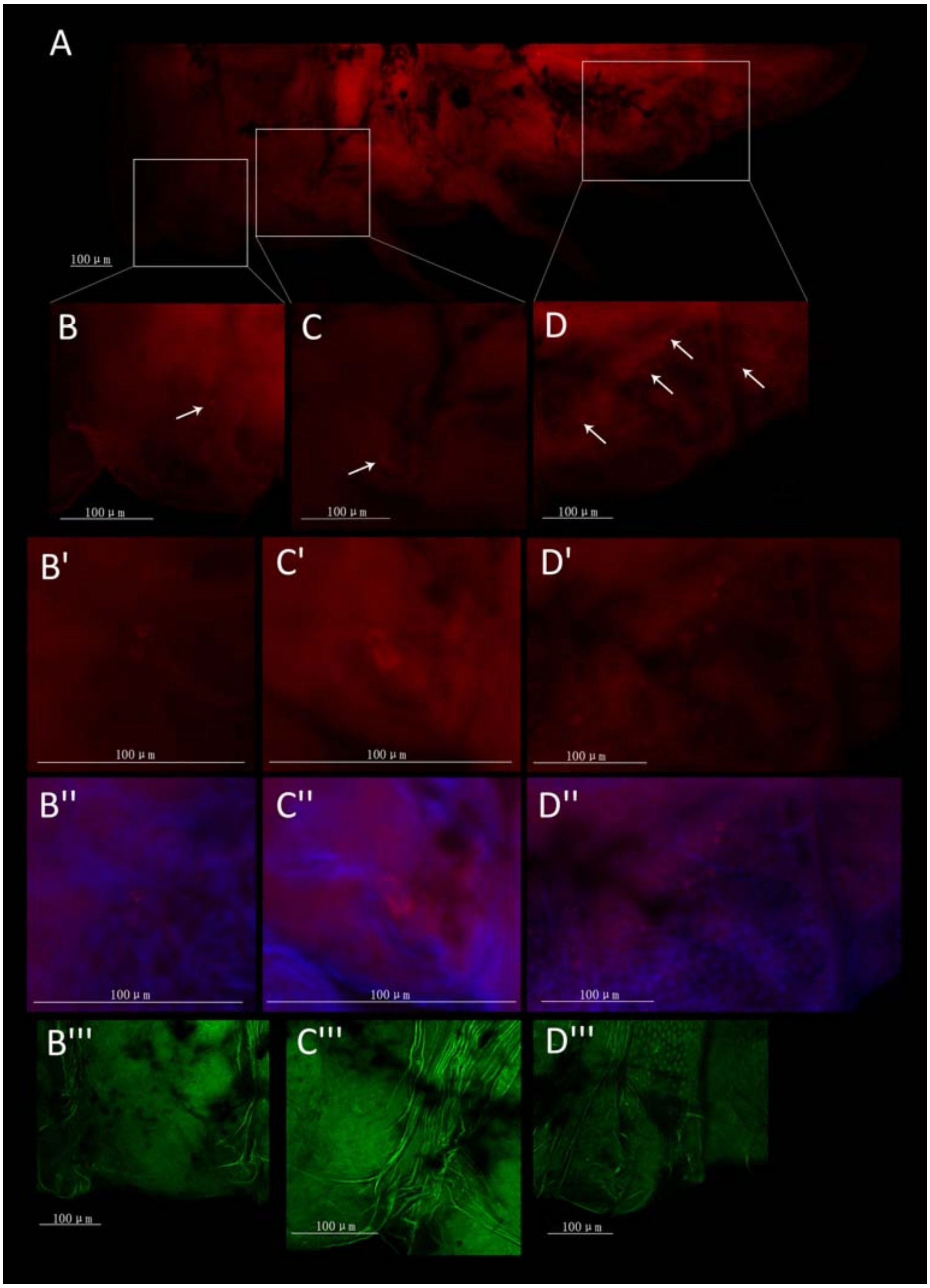

Supplementary Figure 19 *Abd-A* localization in the abdomen of J1 larva of *E.*

sinensis. The *Abd-A* mRNA primarily located in the second, third, sixth ventral segment and the caudal segment (arrows). The result showed the weaker positive signal than LM larva. A. Abdomen of J1 larva; B. The second ventral segment of J1 larva; C. The third ventral segment of J1 larva; D. The sixth ventral segment and the caudal segment of J1 larva; B'-D'. Figure B-D amplification; B''-D''. Merge for 5-FAM and DAPI stained picture; B'''-D'''. Control group with GFP mRNA probe； arrows indicate positive position; scale table shows 100 μM. This experiment was repeated three times independently with similar results.

```
comp57255_c1_seq19  CCCCCCGCCGACACACGCCGCTCATGGTTTATAATGCACAATTTATATAGCGAAAATATCCATAATAATTTGGCCGCAAAAAAGAGTTTCAAGTCTATATTTGGGTTCTTCAAGAGCAAC 120
comp57255_c1_seq23  CCCCCCGCCGACACACGCCGCTCATGGTTTATAATGCACAATTTATATAGCGAAAATATCCATAATAATTTGGCCGCAAAAAAGAGTTTCAAGTCTATATTTGGGTTCTTCAAGAGCAAC 120
comp57255_c1_seq12  CCCCCCGCCGACACACGCCGCTCATGGTTTATAATGCACAATTTATATAGCGAAAATATCCATAATAATTTGGCCGCAAAAAAGAGTTTCAAGTCTATATTTGGGTTCTTCAAGAGCAAC 120
comp57255_c1_seq26  CCCCCCGCCGACACACGCCGCTCATGGTTTATAATGCACAATTTATATAGCGAAAATATCCATAATAATTTGGCCGCAAAAAAGAGTTTCAAGTCTATATTTGGGTTCTTCAAGAGCAAC 120
Consensus           ************************************************************************************************************************ 120

comp57255_c1_seq19  TCACATGTAAGCCCGGAATGCAGAGGGCGGAGCCTGCGGGAAGTGGGCGGGCGTGGGGCGGAGCGTGATTGGCCGGTGGGCGGGTCGAGGGCGAGGCGCTGTGCGTGGCTCCTCCCCCGG 240
comp57255_c1_seq23  TCACATGTAAGCCCGGAATGCAGAGGGCGGAGCCTGCGGGAAGTGGGCGGGCGTGGGGCGGAGCGTGATTGGCCGGTGGGCGGGTCGAGGGCGAGGCGCTGTGCGTGGCTCCTCCCCCGG 240
comp57255_c1_seq12  TCACATGTAAGCCCGGAATGCAGAGGGCGGAGCCTGCGGGAAGTGGGCGGGCGTGGGGCGGAGCGTGATTGGCCGGTGGGCGGGTCGAGGGCGAGGCGCTGTGCGTGGCTCCTCCCCCGG 240
comp57255_c1_seq26  TCACATGTAAGCCCGGAATGCAGAGGGCGGAGCCTGCGGGAAGTGGGCGGGCGTGGGGCGGAGCGTGATTGGCCGGTGGGCGGGTCGAGGGCGAGGCGCTGTGCGTGGCTCCTCCCCCGG 240
Consensus           ************************************************************************************************************************ 240

comp57255_c1_seq19  CGTAGGTGCGCAGTGCCCAGGGGCGGTGCTCGTGACAGCTCATTCATTTGTGGCGGCGGCCGGCGTGATTGACAGCAGTTGGGGTCCAGTGACAGGCGGACACGCACGCCGCTCCGCTCT 360
comp57255_c1_seq23  CGTAGGTGCGCAGTGCCCAGGGGCGGTGCTCGTGACAGCTCATTCATTTGTGGCGGCGGCCGGCGTGATTGACAGCAGTTGGGGTCCAGTGACAGGCGGACACGCACGCCGCTCCGCTCT 360
comp57255_c1_seq12  CGTAGGTGCGCAGTGCCCAGGGGCGGTGCTCGTGACAGCTCATTCATTTGTGGCGGCGGCCGGCGTGATTGACAGCAGTTGGGGTCCAGTGACAGGCGGACACGCACGCCGCTCCGCTCT 360
comp57255_c1_seq26  CGTAGGTGCGCAGTGCCCAGGGGCGGTGCTCGTGACAGCTCATTCATTTGTGGCGGCGGCCGGCGTGATTGACAGCAGTTGGGGTCCAGTGACAGGCGGACACGCACGCCGCTCCGCTCT 360
Consensus           ************************************************************************************************************************ 360

comp57255_c1_seq19  CAGTCGCCTCATGTCGATTGACGTGTTGACGCGCGGTGCAGGTACGGCGCGGTGCTAGACGGTGCAGTGCGGTGCGCGGGTGGGCGTGTAGTGACTGTTGCGGCGCGACATTGTGGTGGT 480
comp57255_c1_seq23  CAGTCGCCTCATGTCGATTGACGTGTTGACGCGCGGTGCAGGTACGGCGCGGTGCTAGACGGTGCAGTGCGGTGCGCGGGTGGGCGTGTAGTGACTGTTGCGGCGCGACATTGTGGTGGT 480
comp57255_c1_seq12  CAGTCGCCTCATGTCGATTGACGTGTTGACGCGCGGTGCAGGTACGGCGCGGTGCTAGACGGTGCAGTGCGGTGCGCGGGTGGGCGTGTAGTGACTGTTGCGGCGCGACATTGTGGTGGT 480
comp57255_c1_seq26  CAGTCGCCTCATGTCGATTGACGTGTTGACGCGCGGTGCAGGTACGGCGCGGTGCTAGACGGTGCAGTGCGGTGCGCGGGTGGGCGTGTAGTGACTGTTGCGGCGCGACATTGTGGTGGT 480
Consensus           ************************************************************************************************************************ 480

comp57255_c1_seq19  GTACCGAGTGAAGCAAGCACAGTACTCTTGGGTGACATGGCTCAGCCTGCCAGGTACGACGACGCGCTGGGGCACTACGGCGGCGGCGGCACGAACATGGAGACCGTAGCCAACATGTAC 600
comp57255_c1_seq23  GTACCGAGTGAAGCAAGCACAGTACTCTTGGGTGACATGGCTCAGCCTGCCAGGTACGACGACGCGCTGGGGCACTACGGCGGCGGCGGCACGAACATGGAGACCGTAGCCAACATGTAC 600
comp57255_c1_seq12  GTACCGAGTGAAGCAAGCACAGTACTCTTGGGTGACATGGCTCAGCCTGCCAGGTACGACGACGCGCTGGGGCACTACGGCGGCGGCGGCACGAACATGGAGACCGTAGCCAACATGTAC 600
comp57255_c1_seq26  GTACCGAGTGAAGCAAGCACAGTACTCTTGGGTGACATGGCTCAGCCTGCCAGGTACGACGACGCGCTGGGGCACTACGGCGGCGGCGGCACGAACATGGAGACCGTAGCCAACATGTAC 600
Consensus           ************************************************************************************************************************ 600

comp57255_c1_seq19  GGCGACCCTCACCGCAGCGCGGCGGCGGCGGCGGCCCTACAGCCGTCCCTCATAAACCACATGAACCACGTGGGCGGGGCCGCCCACAATGCAGCCATGTACGCCCATACACAAGCCCCA 720
comp57255_c1_seq23  GGCGACCCTCACCGCAGCGCGGCGGCGGCGGCGGCCCTACAGCCGTCCCTCATAAACCACATGAACCACGTGGGCGGGGCCGCCCACAATGCAGCCATGTACGCCCATACACAAGCCCCA 720
comp57255_c1_seq12  GGCGACCCTCACCGCAGCGCGGCGGCGGCGGCGGCCCTACAGCCGTCCCTCATAAACCACATGAACCACGTGGGCGGGGCCGCCCACAATGCAGCCATGTACGCCCATACACAAGCCCCA 720
comp57255_c1_seq26  GGCGACCCTCACCGCAGCGCGGCGGCGGCGGCGGCCCTACAGCCGTCCCTCATAAACCACATGAACCACGTGGGCGGGGCCGCCCACAATGCAGCCATGTACGCCCATACACAAGCCCCA 720
Consensus           ************************************************************************************************************************ 720

comp57255_c1_seq19  AACCATGTGGCGGCGAATCATGTGATGGGCTCCGTTCCCGACGTGCACAAACGCGACAAAGACCTCATATACGGGCATCCGCTGTTCCCGCTCCTGGCGCTGATCTTCGAGAAATGCGAG 840
comp57255_c1_seq23  AACCATGTGGCGGCGAATCATGTGATGGGCTCCGTTCCCGACGTGCACAAACGCGACAAAGACCTCATATACGGGCATCCGCTGTTCCCGCTCCTGGCGCTGATCTTCGAGAAATGCGAG 840
comp57255_c1_seq12  AACCATGTGGCGGCGAATCATGTGATGGGCTCCGTTCCCGACGTGCACAAACGCGACAAAGACCTCATATACGGGCATCCGCTGTTCCCGCTCCTGGCGCTGATCTTCGAGAAATGCGAG 840
comp57255_c1_seq26  AACCATGTGGCGGCGAATCATGTGATGGGCTCCGTTCCCGACGTGCACAAACGCGACAAAGACCTCATATACGGGCATCCGCTGTTCCCGCTCCTGGCGCTGATCTTCGAGAAATGCGAG 840
Consensus           ************************************************************************************************************************ 840

comp57255_c1_seq19  TTGGCCACATGTACGCCGCGGGAGCCGGGGGTGGCTGGCGGCGACGTCTGCTCCTCAGAATCCTTCAACGAGGACATCGCCGTCTTTTCCAAACAGCTTCGTCATGAGAAGCCCTACTAC 960
comp57255_c1_seq23  TTGGCCACATGTACGCCGCGGGAGCCGGGGGTGGCTGGCGGCGACGTCTGCTCCTCAGAATCCTTCAACGAGGACATCGCCGTCTTTTCCAAACAGCTTCGTCATGAGAAGCCCTACTAC 960
comp57255_c1_seq12  TTGGCCACATGTACGCCGCGGGAGCCGGGGGTGGCTGGCGGCGACGTCTGCTCCTCAGAATCCTTCAACGAGGACATCGCCGTCTTTTCCAAACAGCTTCGTCATGAGAAGCCCTACTAC 960
comp57255_c1_seq26  TTGGCCACATGTACGCCGCGGGAGCCGGGGGTGGCTGGCGGCGACGTCTGCTCCTCAGAATCCTTCAACGAGGACATCGCCGTCTTTTCCAAACAGCTTCGTCATGAGAAGCCCTACTAC 960
Consensus           ************************************************************************************************************************ 960

comp57255_c1_seq19  TACTCACATCCAGAAGTTGACAGCATGATGGTTCAAGCAATTCAGGTCCTCCGGTTTCACCTCTTAGAGTTAGAAAAAGTGCACGAGCTGTGTGACAACTTCTGCCACCGGTACATATCG 1080
comp57255_c1_seq23  TACTCACATCCAGAAGTTGACAGCATGATGGTTCAAGCAATTCAGGTCCTCCGGTTTCACCTCTTAGAGTTAGAAAAAGTGCACGAGCTGTGTGACAACTTCTGCCACCGGTACATATCG 1080
comp57255_c1_seq12  TACTCACATCCAGAAGTTGACAGCATGATGGTTCAAGCAATTCAGGTCCTCCGGTTTCACCTCTTAGAGTTAGAAAAAGTGCACGAGCTGTGTGACAACTTCTGCCACCGGTACATATCG 1080
comp57255_c1_seq26  TACTCACATCCAGAAGTTGACAGCATGATGGTTCAAGCAATTCAGGTCCTCCGGTTTCACCTCTTAGAGTTAGAAAAAGTGCACGAGCTGTGTGACAACTTCTGCCACCGGTACATATCG 1080
Consensus           ************************************************************************************************************************ 1080

comp57255_c1_seq19  TGTTTGAAGGGCAAGATGCCCATCGATTTGGTCATTGACGAGAGGGACGGCCCCAAGCCCGACCTGGGCACTGGGGACTCAAACAACAACGCCGCCGCCGCCGCTGCCGGGCGGGCCTCG 1200
comp57255_c1_seq23  TGTTTGAAGGGCAAGATGCCCATCGATTTGGTCATTGACGAGAGGGACGGCCCCAAGCCCGACCTGGGCACTGGGGACTCAAACAACAACGCCGCCGCCGCCGCTGCCGGGCGGGCCTCG 1200
comp57255_c1_seq12  TGTTTGAAGGGCAAGATGCCCATCGATTTGGTCATTGACGAGAGGGACGGCCCCAAGCCCGACCTGGGCACTGGGGACTCAAACAACAACGCCGCCGCCGCCGCTGCCGGGCGGGCCTCG 1200
comp57255_c1_seq26  TGTTTGAAGGGCAAGATGCCCATCGATTTGGTCATTGACGAGAGGGACGGCCCCAAGCCCGACCTGGGCACTGGGGACTCAAACAACAACGCCGCCGCCGCCGCTGCCGGGCGGGCCTCG 1200
Consensus           ************************************************************************************************************************ 1200

comp57255_c1_seq19  GCCGACACCACGCACACCGACGGCGCCTCCACTCCTGAC--------------------------------------------------------------------------------- 1239
comp57255_c1_seq23  GCCGACACCACGCACACCGACGGCGCCTCCACTCCTGAC--------------------------------------------------------------------------------- 1239
comp57255_c1_seq12  GCCGACACCACGCACACCGACGGCGCCTCCACTCCTGACGTGGGTCCCTCGTGGGATTCGAGTGTTGACTCTAAGGGAGTCCCCAAACCCGACCAGCCTGAATTCGCATCATCGGCGCTG 1320
comp57255_c1_seq26  GCCGACACCACGCACACCGACGGCGCCTCCACTCCTGACGTGGGTCCCTCGTGGGATTCGAGTGTTGACTCTAAGGGAGTCCCCAAACCCGACCAGCCTGAATTCGCATCATCGGCGCTG 1320
Consensus           ***************************************                                                                                  1239

comp57255_c1_seq19  ---------------CGGCCGCCGTCTTCGTCAATGTCGTATTCTGGGGGTGCGGGCGGGGAGGACGCCCGATCCCCAGGGTCGGGCGGCACCCCGGGTCCCCTCTCCCACCCGCCCGCC 1344
comp57255_c1_seq23  ---------------CGGCCGCCGTCTTCGTCAATGTCGTATTCTGGGGGTGCGGGCGGGGAGGACGCCCGATCCCCAGGGTCGGGCGGCACCCCGGGTCCCCTCTCCCACCCGCCCGCC 1344
comp57255_c1_seq12  CTCTGTATGCCAAAGCGGCCGCCGTCTTCGTCAATGTCGTATTCTGGGGGTGCGGGCGGGGAGGACGCCCGATCCCCAGGGTCGGGCGGCACCCCGGGTCCCCTCTCCCACCCGCCCGCC 1440
comp57255_c1_seq26  CTCTGTATGCCAAAGCGGCCGCCGTCTTCGTCAATGTCGTATTCTGGGGGTGCGGGCGGGGAGGACGCCCGATCCCCAGGGTCGGGCGGCACCCCGGGTCCCCTCTCCCACCCGCCCGCC 1440
Consensus                          *********************************************************************************************************

comp57255_c1_seq19  TCCCAGCAATCAGGAGACAACACCAGCGAAGCAGTGTTTCCTACGCCAACAGGTGGGTCAGATCCCGCCACTCTAGTACGAGGGATGACAGATTCATTAGGTAAGAAGAGCCCCGCTGGT 1464
comp57255_c1_seq23  TCCCAGCAATCAGGAGACAACACCAGCGAAGCAG------------------GTGGGTCAGATCCCGCCACTCTAGTACGAGGGATGACAGATTCATTAG-------------------- 1428
comp57255_c1_seq12  TCCCAGCAATCAGGAGACAACACCAGCGAAGCAGTGTTTCCTACGCCAACAGGTGGGTCAGATCCCGCCACTCTAGTACGAGGGATGACAGATTCATTAGGTAAGAAGAGCCCCGCTGGT 1560
comp57255_c1_seq26  TCCCAGCAATCAGGAGACAACACCAGCGAAGCAG-------------------------------------------------------------------------------------- 1476
Consensus           **********************************                                                                                       1380

comp57255_c1_seq19  CTACACTCTCGCCTCTTTCATGAGTTTGAAGGTGACGCCAGCAACGCAAGCATCGGCTCAGGGGAAGGCACCGAGGAGGATGACGATGGCAGCAAGAAGAACCAGAAGAAAAGGGGCATC 1584
comp57255_c1_seq23  -------------------------------GTGACGCCAGCAACGCAAGCATCGGCTCAGGGGAAGGCACCGAGGAGGATGACGATGGCAGCAAGAAGAACCAGAAGAAAAGGGGCATC 1515
comp57255_c1_seq12  CTACACTCTCGCCTCTTTCATGAGTTTGAAGGTGACGCCAGCAACGCAAGCATCGGCTCAGGGGAAGGCACCGAGGAGGATGACGATGGCAGCAAGAAGAACCAGAAGAAAAGGGGCATC 1680
comp57255_c1_seq26  -------------------------------GTGACGCCAGCAACGCAAGCATCGGCTCAGGGGAAGGCACCGAGGAGGATGACGATGGCAGCAAGAAGAACCAGAAGAAAAGGGGCATC 1563
Consensus                                          *****************************************************************************************

comp57255_c1_seq19  TTCCCTAAAGTGGCCACCAACATCCTCCGGGCCTGGCTCTTCCAACACCTCACACATCCGTACCCCTCGGAGGACCAGAAGAAACAGCTGGCTCAGGACACGGGCCTAACCATCCTGCAG 1704
comp57255_c1_seq23  TTCCCTAAAGTGGCCACCAACATCCTCCGGGCCTGGCTCTTCCAACACCTCACACATCCGTACCCCTCGGAGGACCAGAAGAAACAGCTGGCTCAGGACACGGGCCTAACCATCCTGCAG 1635
comp57255_c1_seq12  TTCCCTAAAGTGGCCACCAACATCCTCCGGGCCTGGCTCTTCCAACACCTCACACATCCGTACCCCTCGGAGGACCAGAAGAAACAGCTGGCTCAGGACACGGGCCTAACCATCCTGCAG 1800
comp57255_c1_seq26  TTCCCTAAAGTGGCCACCAACATCCTCCGGGCCTGGCTCTTCCAACACCTCACACATCCGTACCCCTCGGAGGACCAGAAGAAACAGCTGGCTCAGGACACGGGCCTAACCATCCTGCAG 1683
Consensus           ************************************************************************************************************************ 1587

comp57255_c1_seq19  GTCAACAACTGGTTTATCAATGCCCGCCGGCGGATAGTTCAGCCGCTCATAGATCAGTCGAACCGTGCGGGAGCCATGCCGCACTTTCCAGGTCCGGCCGGCGCCTACTCCCCCGATGCG 1824
comp57255_c1_seq23  GTCAACAACTGGTTTATCAATGCCCGCCGGCGGATAGTTCAGCCGCTCATAGATCAGTCGAACCGTGCGGGAGCCATGCCGCACTTTCCAGGTCCGGCCGGCGCCTACTCCCCCGATGCG 1755
comp57255_c1_seq12  GTCAACAACTGGTTTATCAATGCCCGCCGGCGGATAGTTCAGCCGCTCATAGATCAGTCGAACCGTGCGGGAGCCATGCCGCACTTTCCAGGTCCGGCCGGCGCCTACTCCCCCGATGCG 1920
comp57255_c1_seq26  GTCAACAACTGGTTTATCAATGCCCGCCGGCGGATAGTTCAGCCGCTCATAGATCAGTCGAACCGTGCGGGAGCCATGCCGCACTTTCCAGGTCCGGCCGGCGCCTACTCCCCCGATGCG 1803
Consensus           ************************************************************************************************************************ 1707

comp57255_c1_seq19  GCGTCCATGGGCTACATGATGGACGGCCAGCAGCACATGTTCCGCGCCTCAGACCCCTACACCGACCATGCCGCCGCCGCCGCCTACTACGGACACCCGGCGGCCGCATACAACTACCCT 1944
comp57255_c1_seq23  GCGTCCATGGGCTACATGATGGACGGCCAGCAGCACATGTTCCGCGCCTCAGACCCCTACACCGACCATGCCGCCGCCGCCGCCTACTACGGACACCCGGCGGCCGCATACAACTACCCT 1875
comp57255_c1_seq12  GCGTCCATGGGCTACATGATGGACGGCCAGCAGCACATGTTCCGCGCCTCAGACCCCTACACCGACCATGCCGCCGCCGCCGCCTACTACGGACACCCGGCGGCCGCATACAACTACCCT 2040
comp57255_c1_seq26  GCGTCCATGGGCTACATGATGGACGGCCAGCAGCACATGTTCCGCGCCTCAGACCCCTACACCGACCATGCCGCCGCCGCCGCCTACTACGGACACCCGGCGGCCGCATACAACTACCCT 1923
Consensus           ************************************************************************************************************************ 1827

comp57255_c1_seq19  CATCATCTATAGGTCCGCGTGTAGCGGTCAAGTAGCGTGAGCGTGGTGTCCCCGATGCCCTGAGCCACGAGCAGCCCCGCGCCGAGTCCCCAGTGGTGTAGTGTGGCGGCCGCCGGCGAC 2064
comp57255_c1_seq23  CATCATCTATAGGTCCGCGTGTAGCGGTCAAGTAGCGTGAGCGTGGTGTCCCCGATGCCCTGAGCCACGAGCAGCCCCGCGCCGAGTCCCCAGTGGTGTAGTGTGGCGGCCGCCGGCGAC 1995
comp57255_c1_seq12  CATCATCTATAGGTCCGCGTGTAGCGGTCAAGTAGCGTGAGCGTGGTGTCCCCGATGCCCTGAGCCACGAGCAGCCCCGCGCCGAGTCCCCAGTGGTGTAGTGTGGCGGCCGCCGGCGAC 2160
comp57255_c1_seq26  CATCATCTATAGGTCCGCGTGTAGCGGTCAAGTAGCGTGAGCGTGGTGTCCCCGATGCCCTGAGCCACGAGCAGCCCCGCGCCGAGTCCCCAGTGGTGTAGTGTGGCGGCCGCCGGCGAC 2043
Consensus           ************************************************************************************************************************ 1947

comp57255_c1_seq19  AGACAGGACTGTGTGCCCCGCCGGCGGCCGGTGCCCCGCGGCGCGCGTGCCCCGCGCGGCGGCGGCGTCAGGGGAACTGTGTACATATTCTTGGTGTCTCGCGCGTTTACGTTTGTGAGG 2184
comp57255_c1_seq23  AGACAGGACTGTGTGCCCCGCCGGCGGCCGGTGCCCCGCGGCGCGCGTGCCCCGCGCGGCGGCGGCGTCAGGGGAACTGTGTACATATTCTTGGTGTCTCGCGCGTTTACGTTTGTGAGG 2115
comp57255_c1_seq12  AGACAGGACTGTGTGCCCCGCCGGCGGCCGGTGCCCCGCGGCGCGCGTGCCCCGCGCGGCGGCGGCGTCAGGGGAACTGTGTACATATTCTTGGTGTCTCGCGCGTTTACGTTTGTGAGG 2280
comp57255_c1_seq26  AGACAGGACTGTGTGCCCCGCCGGCGGCCGGTGCCCCGCGGCGCGCGTGCCCCGCGCGGCGGCGGCGTCAGGGGAACTGTGTACATATTCTTGGTGTCTCGCGCGTTTACGTTTGTGAGG 2163
Consensus           ************************************************************************************************************************ 2067

comp57255_c1_seq19  ACTGGCAGGGGTGAGGACGGTGGCGCGGCCTGGGCAGGGGCGTGGCAGGGCGGGGCCGGGCAGGGCATGACACCCTCCCCGCCGCCGCCGCTACTGTTGTGGCTCATCATCATCATCATC 2304
comp57255_c1_seq23  ACTGGCAGGGGTGAGGACGGTGGCGCGGCCTGGGCAGGGGCGTGGCAGGGCGGGGCCGGGCAGGGCATGACACCCTCCCCGCCGCCGCCGCTACTGTTGTGGCTCATCATCATCATCATC 2235
comp57255_c1_seq12  ACTGGCAGGGGTGAGGACGGTGGCGCGGCCTGGGCAGGGGCGTGGCAGGGCGGGGCCGGGCAGGGCATGACACCCTCCCCGCCGCCGCCGCTACTGTTGTGGCTCATCATCATCATCATC 2400
comp57255_c1_seq26  ACTGGCAGGGGTGAGGACGGTGGCGCGGCCTGGGCAGGGGCGTGGCAGGGCGGGGCCGGGCAGGGCATGACACCCTCCCCGCCGCCGCCGCTACTGTTGTGGCTCATCATCATCATCATC 2283
Consensus           ************************************************************************************************************************ 2187

comp57255_c1_seq19  ACCTCAACACCTCACCACCAGCCTGCGCGGGGCGTCGCTGCAGCAGCGTGACGTCAGAAGCATTGCAGCAGCAGAAGCAGTAACCTCCGAGGGCGTCACAAGGACCAGAGCACCGGGAGT 2424
comp57255_c1_seq23  ACCTCAACACCTCACCACCAGCCTGCGCGGGGCGTCGCTGCAGCAGCGTGACGTCAGAAGCATTGCAGCAGCAGAAGCAGTAACCTCCGAGGGCGTCACAAGGACCAGAGCACCGGGAGT 2355
comp57255_c1_seq12  ACCTCAACACCTCACCACCAGCCTGCGCGGGGCGTCGCTGCAGCAGCGTGACGTCAGAAGCATTGCAGCAGCAGAAGCAGTAACCTCCGAGGGCGTCACAAGGACCAGAGCACCGGGAGT 2520
comp57255_c1_seq26  ACCTCAACACCTCACCACCAGCCTGCGCGGGGCGTCGCTGCAGCAGCGTGACGTCAGAAGCATTGCAGCAGCAGAAGCAGTAACCTCCGAGGGCGTCACAAGGACCAGAGCACCGGGAGT 2403
Consensus           ************************************************************************************************************************ 2307

comp57255_c1_seq19  CGCTCCTCCCCGTGACGTCACAACACCACCACCAACACCACCA 2467
comp57255_c1_seq23  CGCTCCTCCCCGTGACGTCACAACACCACCACCAACACCACCA 2398
comp57255_c1_seq12  CGCTCCTCCCCGTGACGTCACAACACCACCACCAACACCACCA 2563
comp57255_c1_seq26  CGCTCCTCCCCGTGACGTCACAACACCACCACCAACACCACCA 2446
Consensus           ******************************************* 2350
```

Supplementary Figure 20. Alignments of nucleotides of four Hth transcripts in *E. sinensis*.

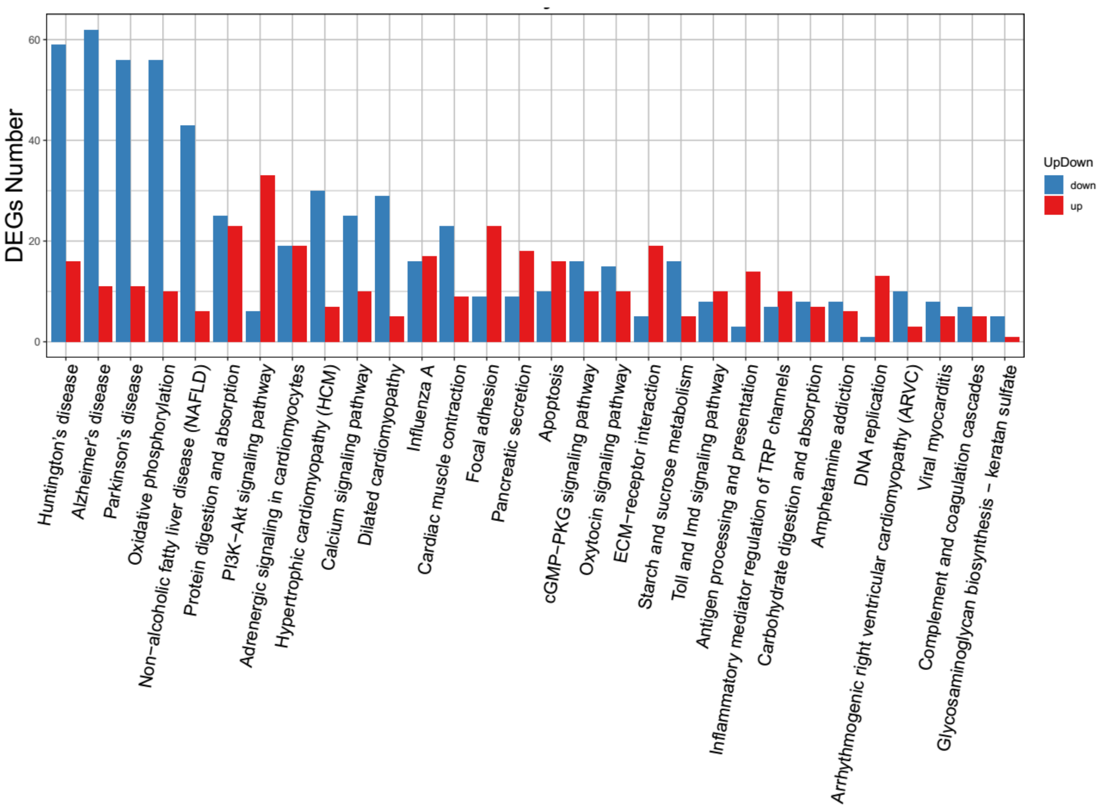

Supplementary Figure 21. The enriched KEGG pathway of differentially expressed genes in the abdomen of *E. sinensis* from the LM to J1 stage.

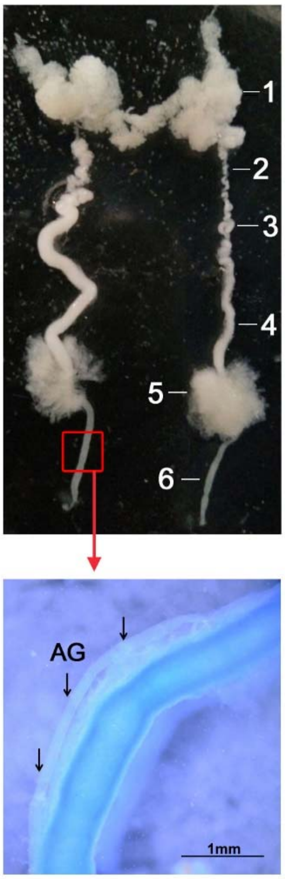

Supplementary Figure 22. Morphology of the male reproductive system structure of *E. sinensis* and microstructure of androgenic gland (AG). 1: testis; 2: vas efferens; 3: vas deferens; 4: seminal vesicle; 5: accessory sex gland; 6: ejaculatory duct. Vas efferens, vas deferens and seminal vesicle are known as the sperm-duct. A pair of AGs is attached to the surface of the ejaculatory duct.

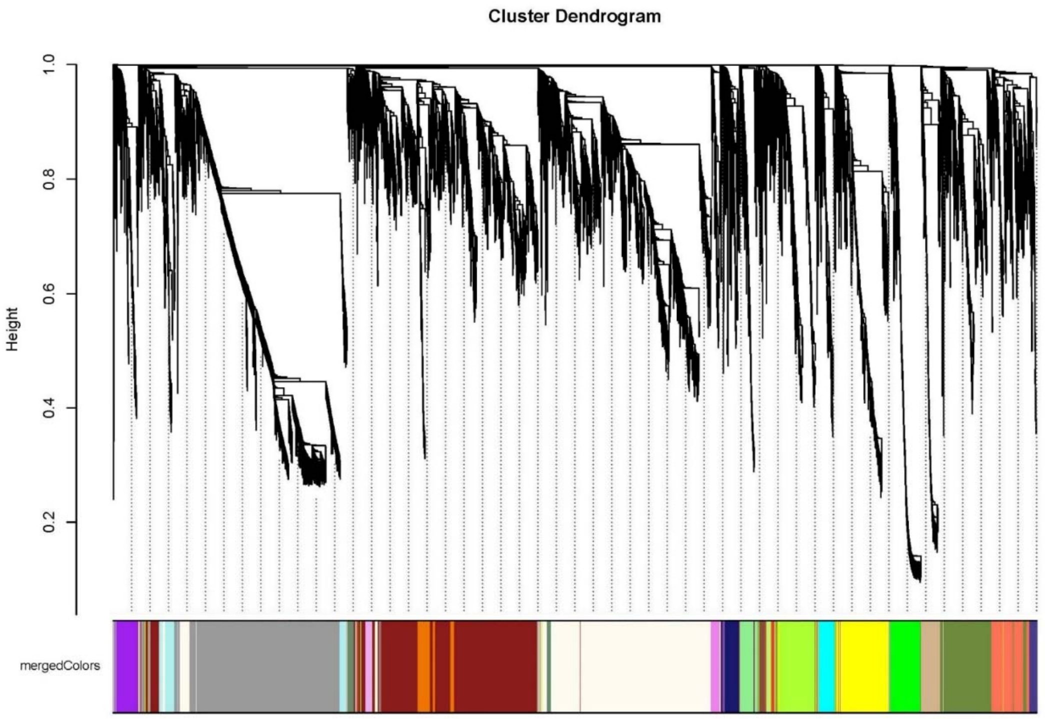

Supplementary Figure 23. Construction of gene co-expression network based on 29 transcriptomes from life history stages and adult tissues. Dendrograms were produced by average linkage hierarchical clustering of genes on the basis of a topological overlap. Horizontal color bars represent different modules of co-expressed genes.

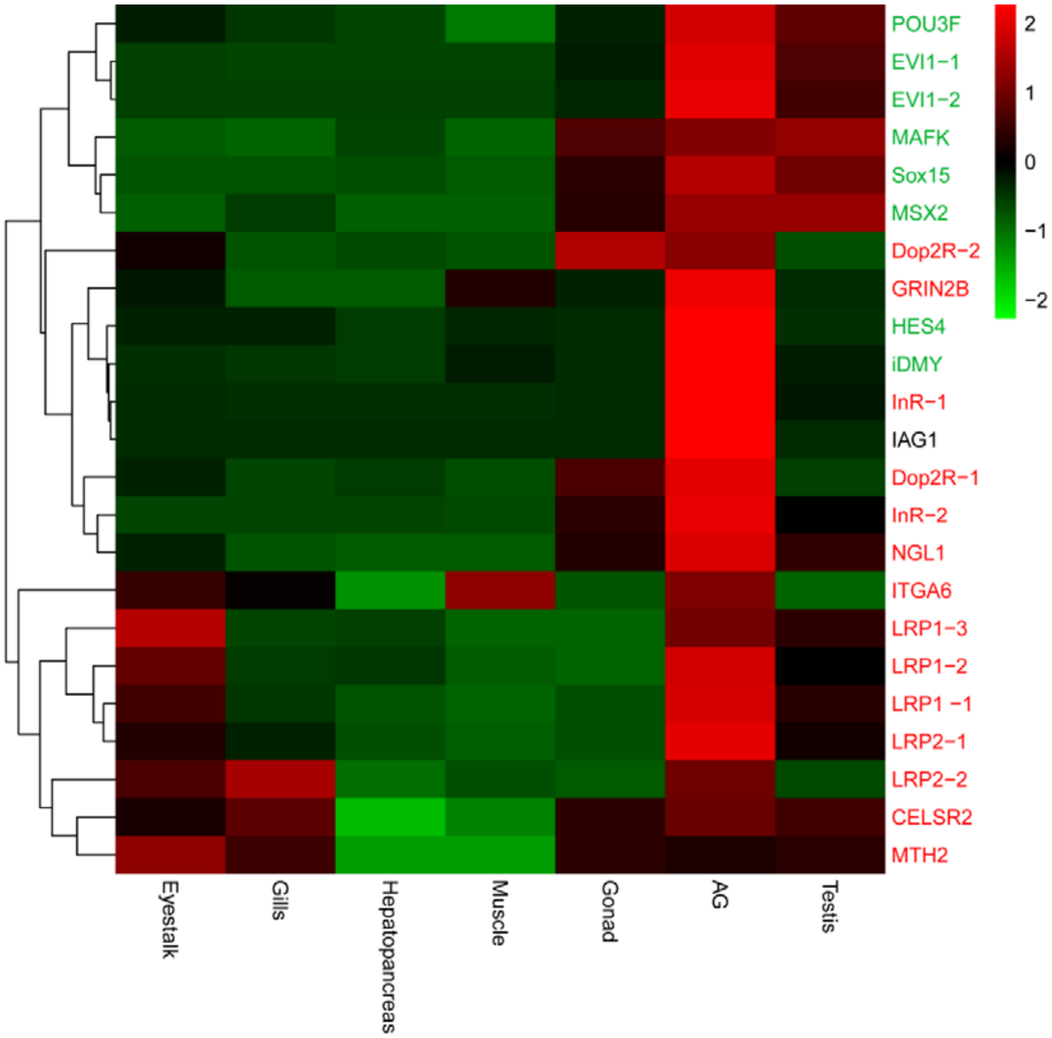

Supplementary Figure 24. The expression heatmap of receptors (red) and transcription factors (green) of the AG-related module in adult tissues of *E. sinensis*.

A

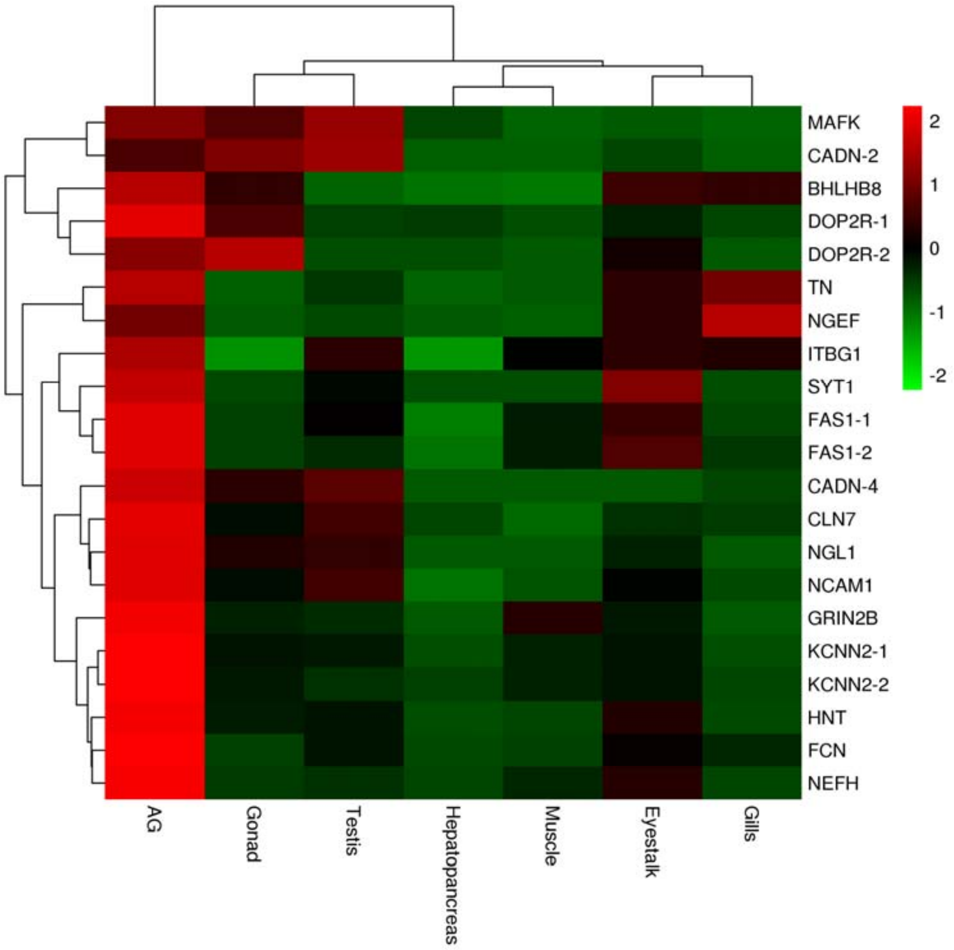

MAFK
CADN-2
BHLHB8
DOP2R-1
DOP2R-2
TN
NGEF
ITBG1
SYT1
FAS1-1
FAS1-2
CADN-4
CLN7
NGL1
NCAM1
GRIN2B
KCNN2-1
KCNN2-2
HNT
FCN
NEFH
2
1
0
-1
-2
AG
Gonad
Testis
Hepatopancreas
Muscle
Eyestalk
Gills

B

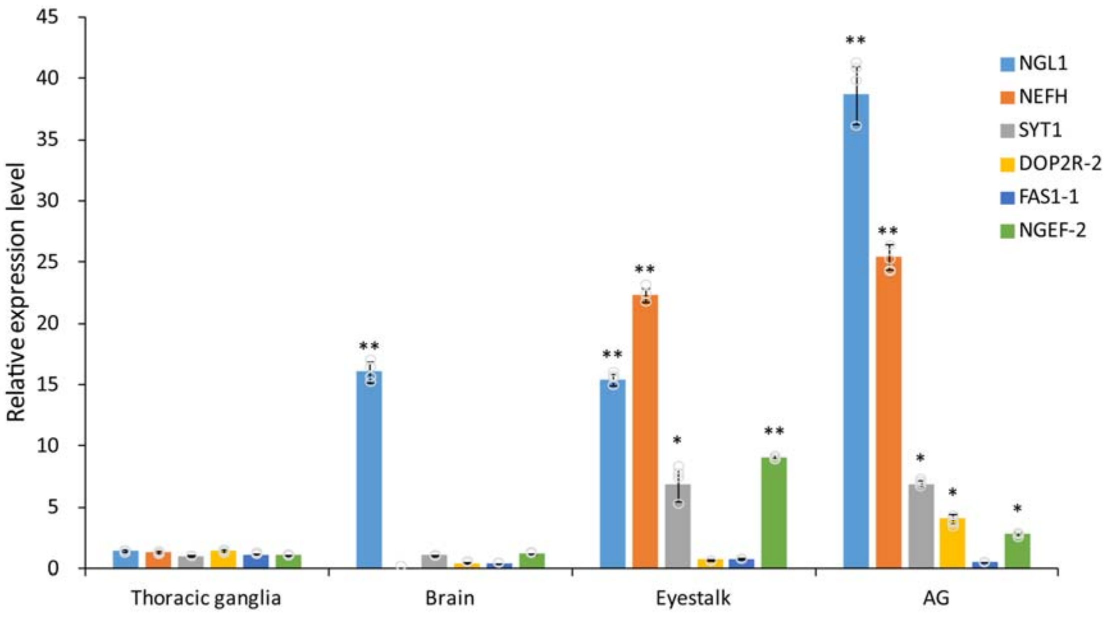

Relative expression level
45
40
35
30
25
20
15
10
5
0
Thoracic ganglia
Brain
Eyestalk
AG
NGL1
NEFH
SYT1
DOP2R-2
FAS1-1
NGEF-2

Supplementary Figure 25. Expression of neuron or axon-related genes from the AG-related module. The expression patterns of these gens in adult tissues (A), and in AG and other nervous tissues qPCR (B). Error bars represent the mean ± S.D. (n = 4). Significant differences across thoracic ganglion tested by two-tailed Student's *t*-test are indicated with an asterisk at $P < 0.05$, and two asterisks at $P < 0.01$. All primer sequences are available in Supplementary Table 21.

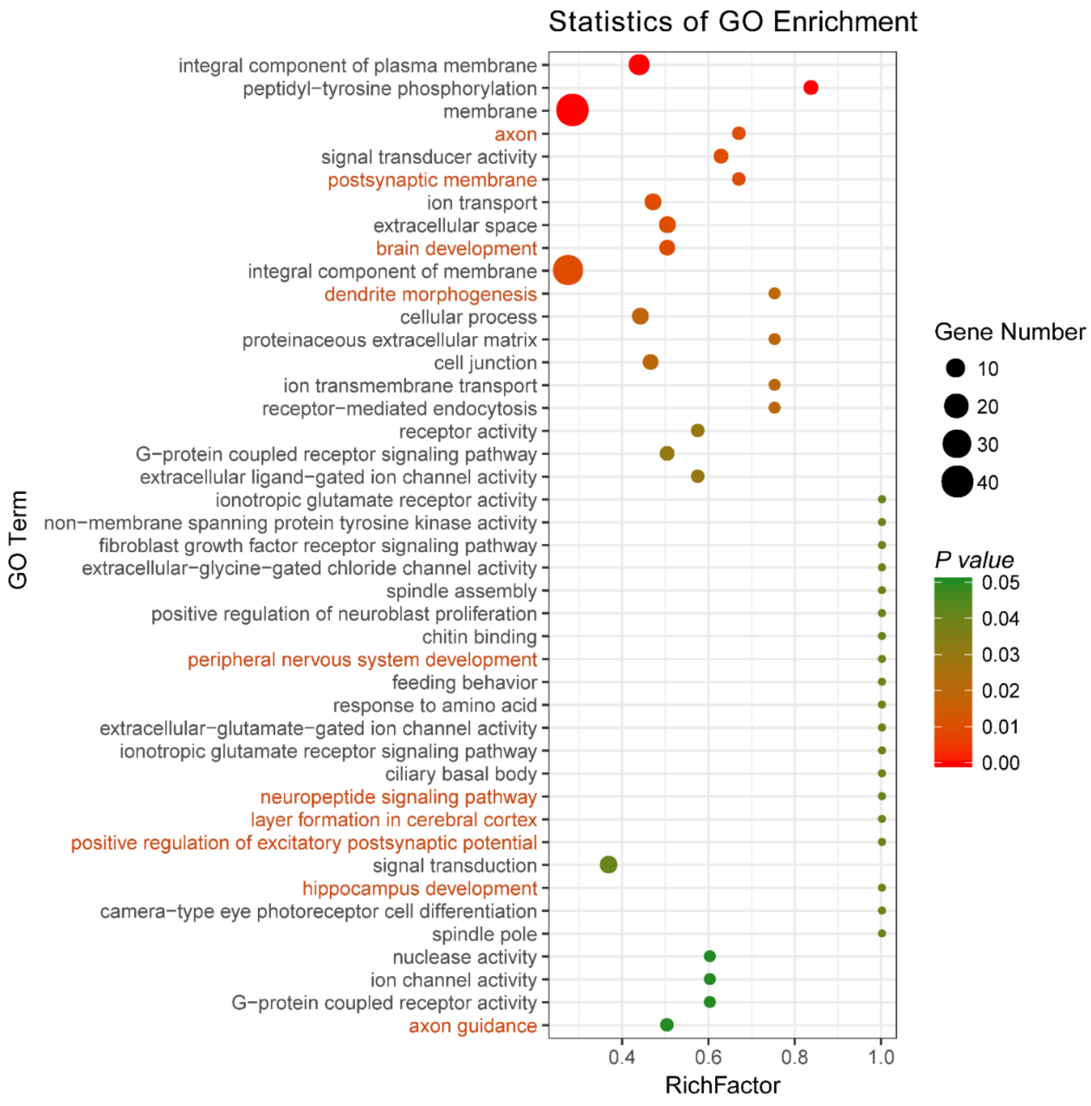

Supplementary Figure 26. GO analysis of the target genes of differently expressed miRNAs in AG between synthesis (SY) and secretion phase (SE). The GO terms related to structures and developmental processes of nervous system are highlighted in orange.

A

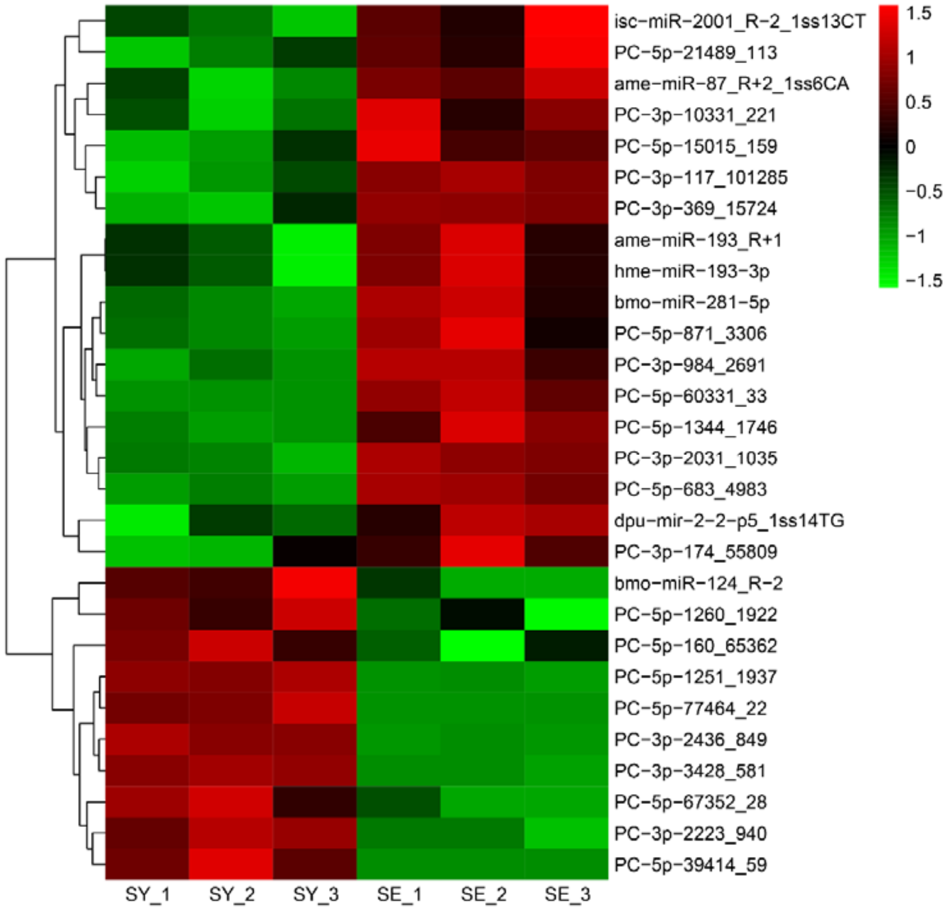
isc-miR-2001_R-2_1ss13CT
PC-5p-21489_113
ame-miR-87_R+2_1ss6CA
PC-3p-10331_221
PC-5p-15015_159
PC-3p-117_101285
PC-3p-369_15724
ame-miR-193_R+1
hme-miR-193-3p
bmo-miR-281-5p
PC-5p-871_3306
PC-3p-984_2691
PC-5p-60331_33
PC-5p-1344_1746
PC-3p-2031_1035
PC-5p-683_4983
dpu-mir-2-2-p5_1ss14TG
PC-3p-174_55809
bmo-miR-124_R-2
PC-5p-1260_1922
PC-5p-160_65362
PC-5p-1251_1937
PC-5p-77464_22
PC-3p-2436_849
PC-3p-3428_581
PC-5p-67352_28
PC-3p-2223_940
PC-5p-39414_59
SY_1
SY_2
SY_3
SE_1
SE_2
SE_3
1.5
1
0.5
0
-0.5
-1
-1.5

B

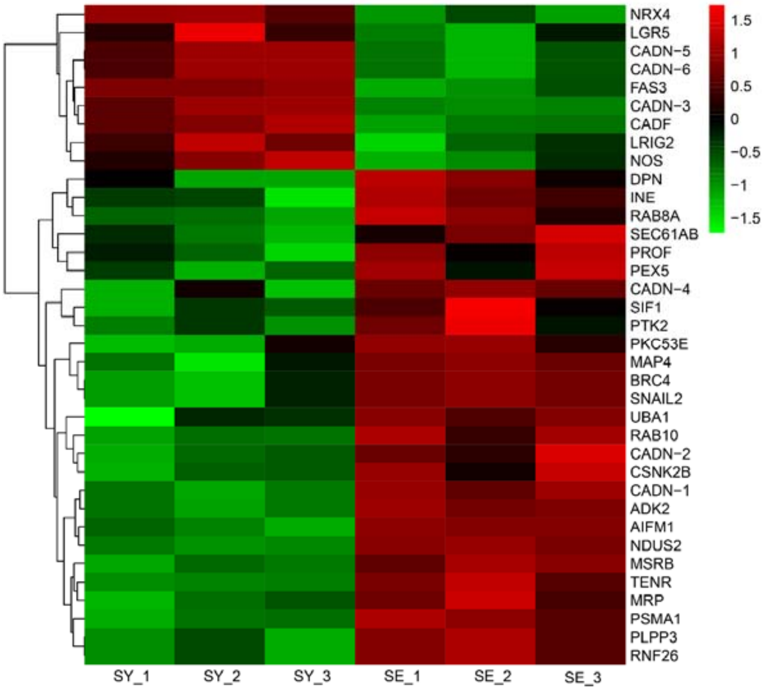
NRX4
LGR5
CADN-5
CADN-6
FAS3
CADN-3
CADF
LRIG2
NOS
DPN
INE
RAB8A
SEC61AB
PROF
PEX5
CADN-4
SIF1
PTK2
PKC53E
MAP4
BRC4
SNAIL2
UBA1
RAB10
CADN-2
CSNK2B
CADN-1
ADK2
AIFM1
NDUS2
MSRB
TENR
MRP
PSMA1
PLPP3
RNF26
SY_1
SY_2
SY_3
SE_1
SE_2
SE_3
1.5
1
0.5
0
-0.5
-1
-1.5

Supplementary Figure 27. Expression profiles of the neuron-related differentially expressed miRNAs (A) and genes (B) between synthesis (SY) and secretion (SE) phase of *E. sinensis*.

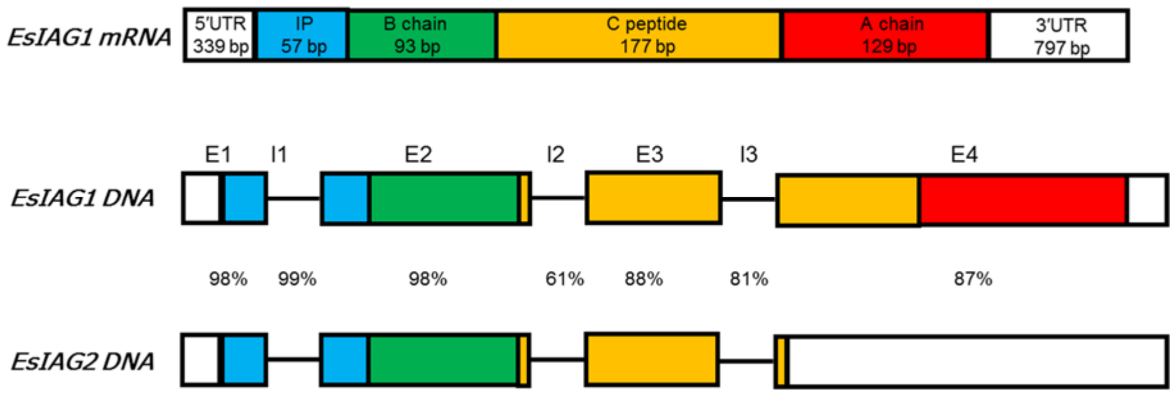

Supplementary Figure 28. Structure of *EsIAG1* mRNA and the genomic structures of *EsIAG1* and *EsIAG2*. The different components of *EsIAG* mRNA are shaded in different colors, and the length of each component is indicated on the figure. Exons and introns of *EsIAG1 DNA and EsIAG2 DNA* are indicated by the letters 'E' and 'I', respectively. The identity (in percentage) of the exons and introns between the two genomic sequences is indicated.

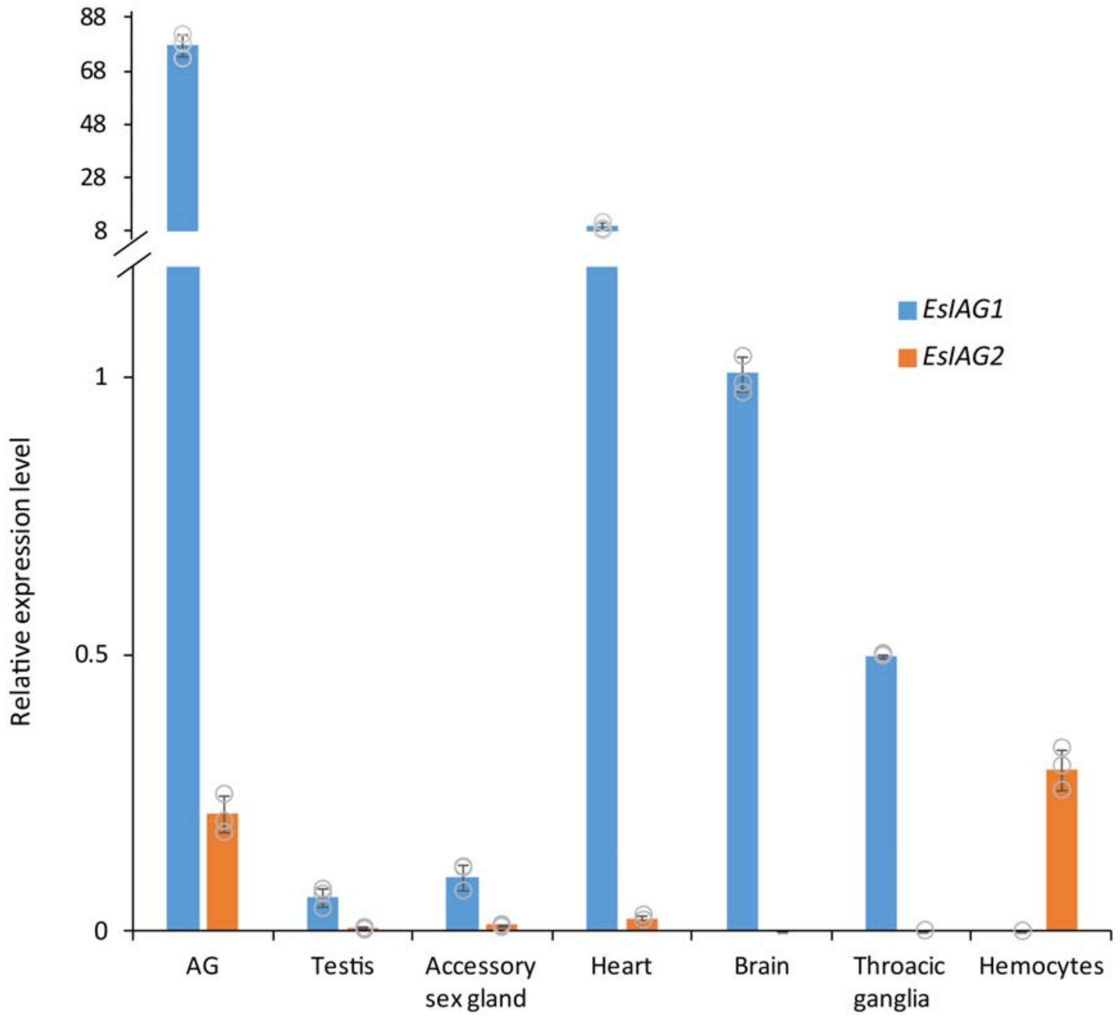

Supplementary Figure 29. Expression pattern of *EsIAG1* and *EsIAG2* in adult tissues based on qPCR. Error bars represent the mean ± S.D. (n = 3). The gene-specific primer sequences are listed in Supplementary Table 21.

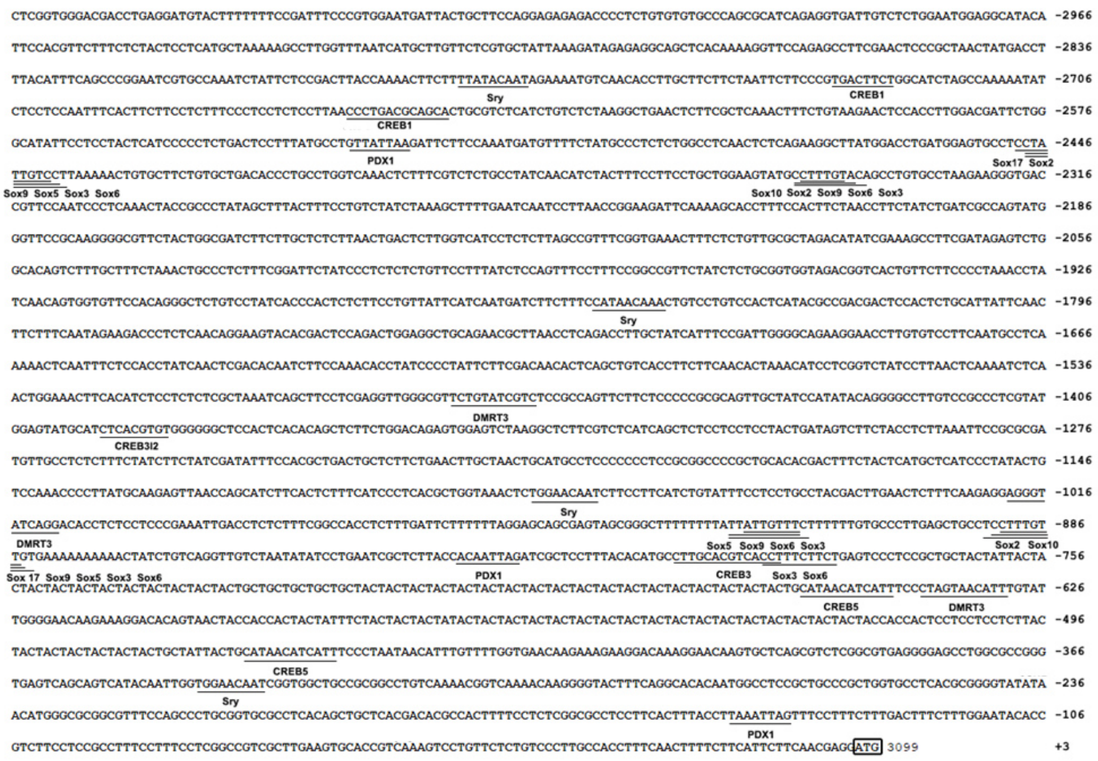

Supplementary Figure 30. Predicted transcription factor binding sites on the 5′-flanking sequence of *EsIAG1*. The sites are underlined with the name of corresponding transcription factors. The start codon is boxed.

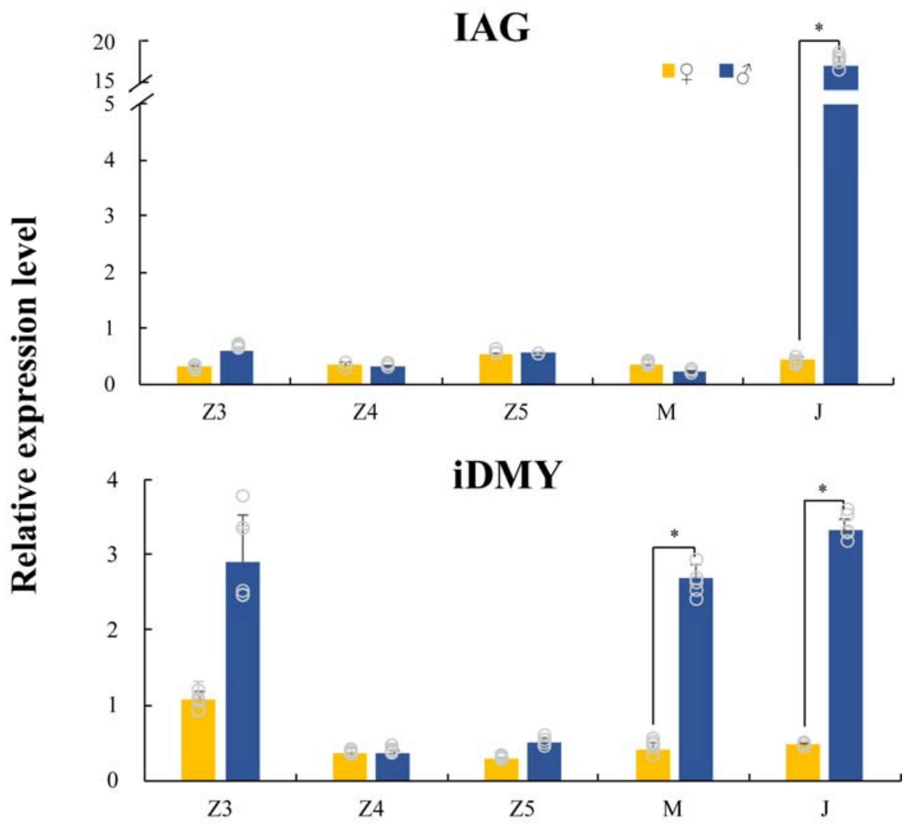

Supplementary Figure 31. The expression pattern of *IAG* and *iDMY* in sex distinguished larva detected by qPCR with primer pairs IAG_RTF/ IAG_RTR and iDMY_RTF/ iDMY_RTR. The sex of larva was determined by two female-specific DNA markers SM_F1/SM_R1 and SM_F2/SM_R2. Error bars represent the mean ± S.D. (n = 5). Significant differences between female and male tested by two-tailed Student's *t*-test are indicated with an asterisk at $P < 0.05$. All primer sequences are available in Supplementary Table 21.

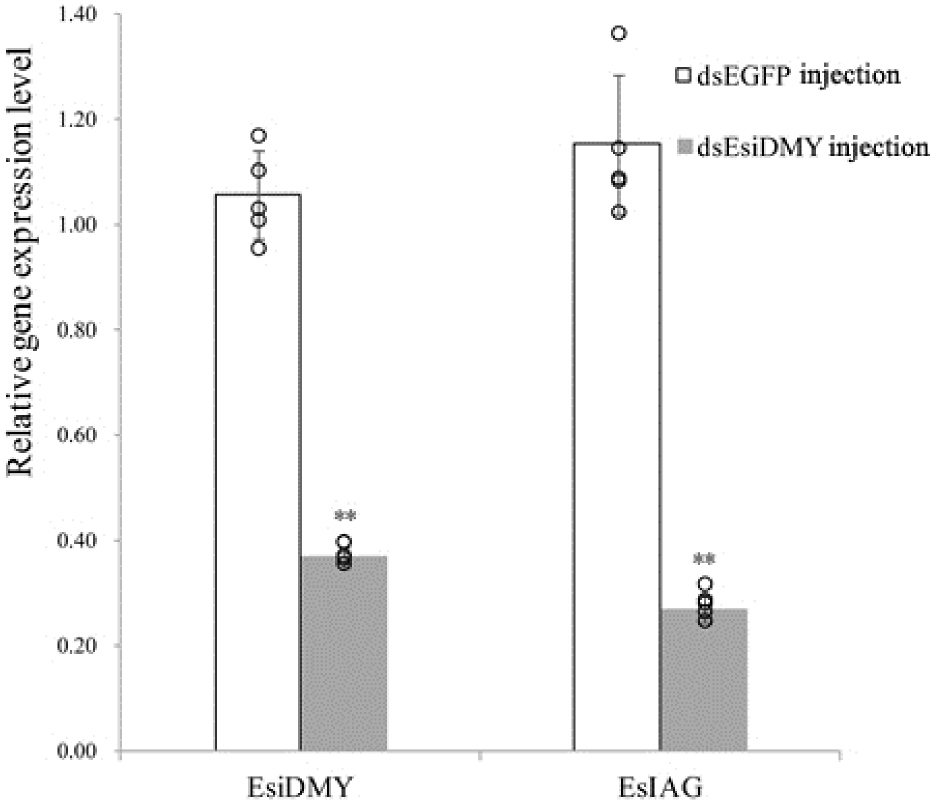

Supplementary Figure 32. The expression pattern of iDMY and IAG in the AG after injection of dsiDMY. 20 crabs were employed for the gene knock-down experiment. They were randomly divided into two groups and each contained 10 individuals. Crabs receiving an injection of dsiDMY dsRNA (2μg/g crab) resuspended in 0.1 mol/L PBS at the arthrodial membrane of the last walking leg were used as challenge group, while the individuals received an injection of EGFP dsRNA (2 μg/g crab) were used as control group. The injected crabs were returned to the water tanks and five individuals were randomly sampled at the time point of 24 h post-injection. Error bars represent the mean ± S.D. (n = 5). Significant differences between two treatments tested by two-tailed Student's *t*-test ($P < 0.01$) are shown with two asterisks (**). All primer sequences are available in Supplementary Table 21.

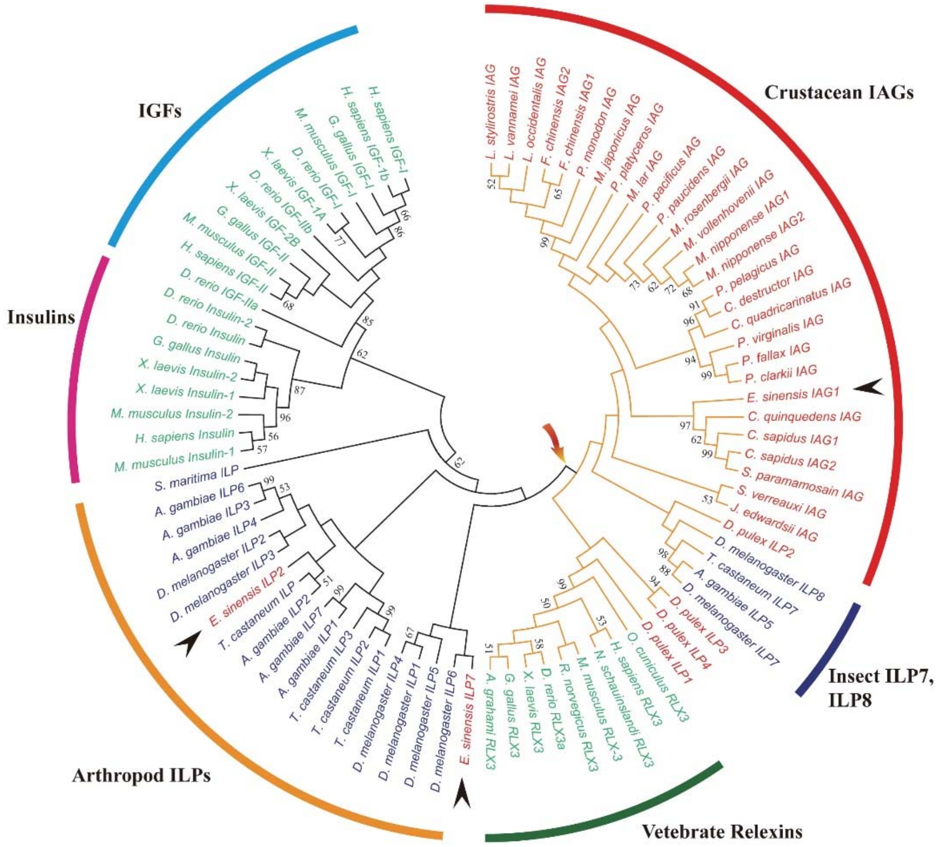

Supplementary Figure 33. Phylogenetic tree inferred from protein sequences of insulin family from representative arthropods and vertebrates inferred by the neighbor-joining (NJ) method. Numbers in each branch indicate bootstrap values above 50%.

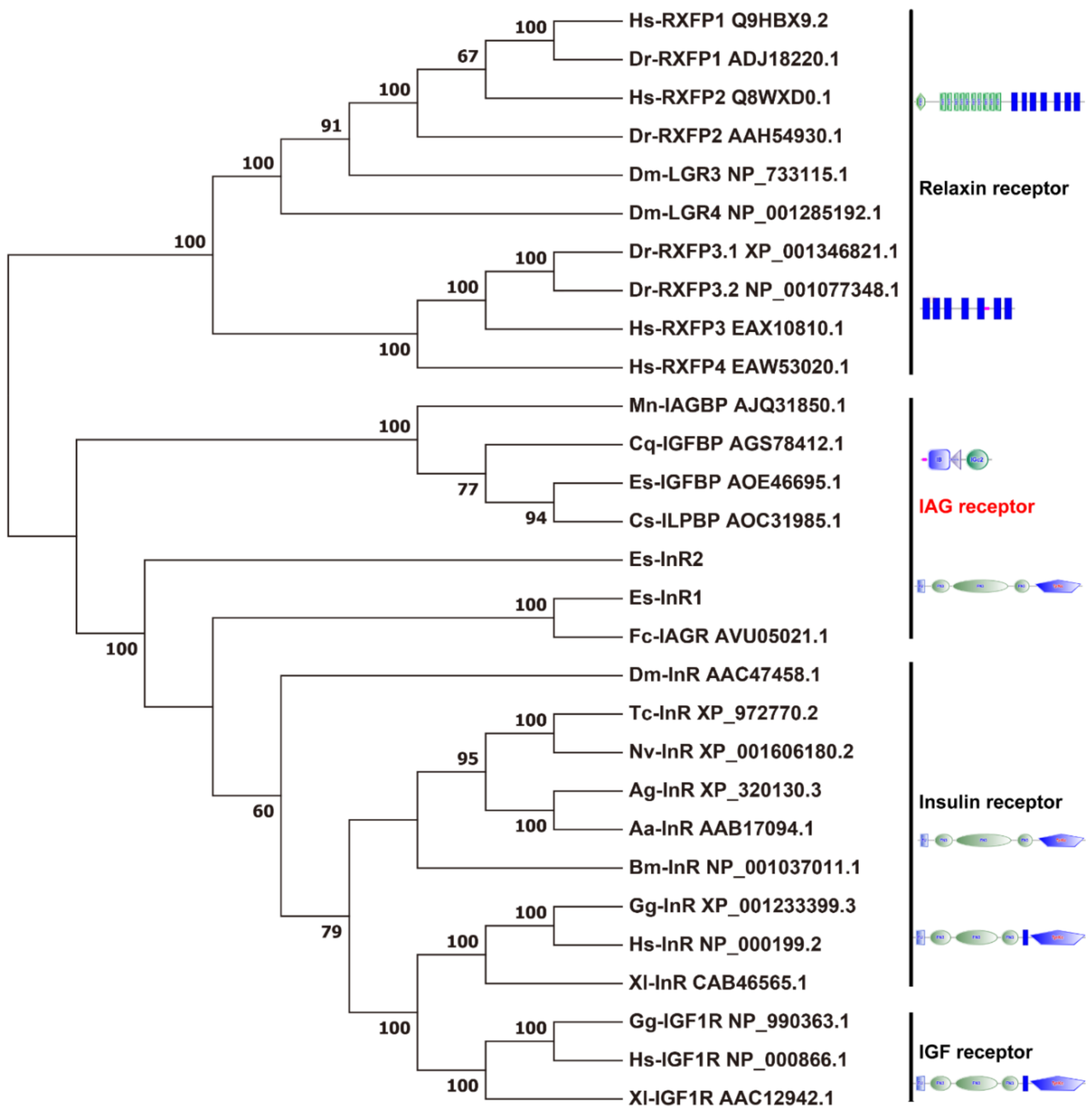

Supplementary Figure 34. Phylogenetic analysis of insulin superfamily member receptors constructed with bootstrap NJ using MEGA 7.0. Numbers represent percent bootstrap values; unlabeled branches indicate a value less than 50. The domains of each cluster were predicted by SMART. RXFP: relaxin receptor; LGR: leucine rich repeat containing G protein coupled receptor; IGFBP: insulin-like growth factor binding protein; ILPBP: insulin-like peptide binding protein; IGF1R: insulin-like growth factor 1 receptor; InR: insulin-like receptor. The species using in phylogenetic

tree: Hs: *Homo sapiens*; Dr: *Danio rerio*; Gg: *Gallus gallus*; Xl: *Xenopus laevis*; Dm: *Drosophila melanogaster*; Tc: *Tribolium castaneum*; Nv: *Nasonia vitripennis*; Ag: *Anopheles gambiae*; Aa: *Aedes aegypti*; Bm: *Bombyx mori*; Fc: *Fenneropenaeus chinensis*; Cs: *Callinectes sapidus*; Cq: *Cherax quadricarinatus*; Mn: *Macrobrachium nipponense*; Es: *Eriocheir sinensis*.

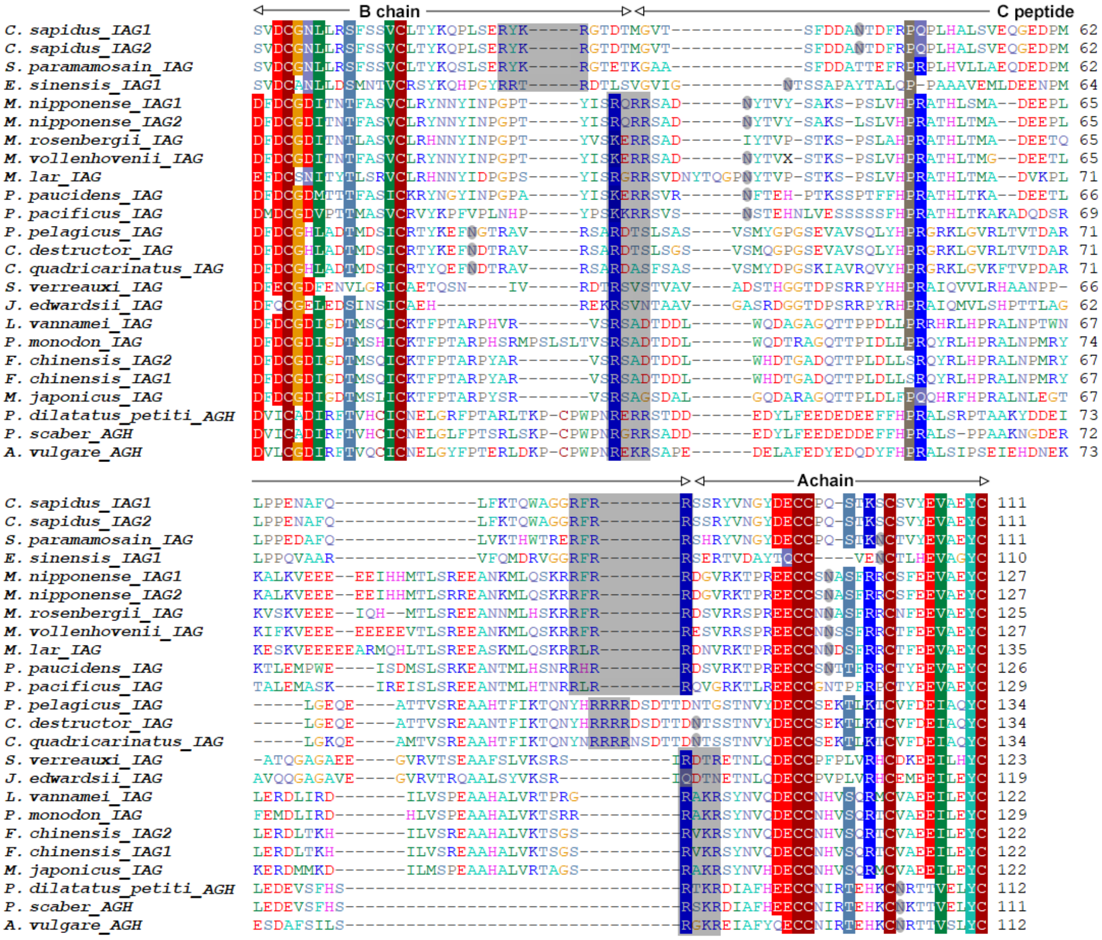

Supplementary Figure 35. Multiple sequence alignment of IAG from *E. sinensis* other species. The identity sequence is shaded with different colors. All IAGs known to date were aligned using CLUSTALX. The predicted cleavage sites (RxxR, KxxR or

xR) are shown with gray box. The most conserved feature is the backbone consisting of six cysteine residues, which give rise to disulfide bridges (black lines, C1-C4, C2-C6, C3-C5 for decapod IAG, and C1-C3, C2-C6, C4-C5 for isopod AGH). Two other cysteine residues that form a second inter-chain disulfide bridge in the isopod species ate also highlighted. N-glycosylation sites with the sequence of NxS/T are highlighted with gray circles.

Supplementary Tables

Supplementary Table 1. Statistics of the genome sequencing data of *E. sinensis*.

	Library	Read length (bp)	Clean data (Gb)	Sequencing depth*	GC (%)
Illumina	250 bp	PE150	68.36	47.14	45.04
	500 bp		128.30	88.48	44.73
	800 bp		101.00	69.66	43.42
	2 kb	PE125	36.11	24.90	45.55
	5 kb		26.64	18.37	46.46
	10 kb		13.75	9.48	49.43
	Total		374.16	258.04	-
PacBio			51.31	35.39	39.00
10×Genomics		PE150	154.10	106.35	48.70

PE: paired-end.

* Based on the estimated genome size of 1.45 Gb according to *k*-mer analysis.

Supplementary Table 2. Summary statistics of the assembled *E. sinensis* genome.

	Allpaths	N50 (bp)	N50 no.	N90 (bp)	N90 no.	Longest (bp)	Total length (bp)
Our assembled genome	Contig	26,045	12,722	2,670	65,176	1,457,336	1,293,881,449
	Scaffold	150,053	2,235	9,448	17,979	50,504,686	1,562,256,418
	Hi-C	N50 (bp)	N50 no.	N90 (bp)	N90 no.	Longest (bp)	Total length (bp)
	Scaffold	17,127,685	30	9,466	7,204	50,864,308	1,567,615,418
Previously published genome by Tang et al.[1]		N50 (bp)	N50 no.	N90 (bp)	N90 no.	Longest (bp)	Total length (bp)
		3,185,988	94	80,260	1,368	16,811,200	1,270,960,592
Previously published genome by Song et al.[2]		N50 (bp)	N50 no.	N90 (bp)	N90 no.	Longest (bp)	Total length (bp)
		111,755	2,066	144	772,162	2,002,076	1,118,179,523

Supplementary Table 3. Assembly statistics of several crustacean genomes.

Species	Class	Order	Chromosome no.	Genome size (bp)	Contig N50 (bp)	Scaffold N50 (bp)
Daphina pulex	Branchiopoda	Diplostraca	2n=24	197,206,209	49,250	642,089
Eulimnadia texana	Branchiopoda	Diplostraca		120,535,642	18,070,303	10,428,323
Eurytemora affinis	Hexanauplia	Calanoida		389,032,277	67,724	252,275
Tigriopus californicus	Hexanauplia	Harpacticoida		191,142,5ds 46	44,438	15,806,032
Hyalella azteca	Malacostraca	Amphipoda		550,885,727	114,415	215,427
Parhyale hawaiensis	Malacostraca	Amphipoda	2n=46	4023,757,376	4,009	69,178
Armadillidium vulgare	Malacostraca	Isopoda	2n=54	1,725,108,002	38,359	51,088
Litopenaeus vannamei	Malacostraca	Decapoda	2n=44	1,663,559,157	57,650	605,555
Eriocheir sinensis	Malacostraca	**Decapoda**	**2n=146**	**1,567,615,418**	**26,045**	**17,127,685**
Portunus trituberculatus	Malacostraca	Decapoda	2n=106	1 005,046,021	4,121,416	21,793,880
Procambarus virginalis	Malacostraca	Decapoda	2n=184	3,290,470,695	1,187	39,275

Supplementary Table 4. Alignment of short reads by BWA.

	PE250	PE500
No. of reads (bp)	577,839,645	597,978,348
No. of mapped reads (bp)	539,641,541	556,923,825
Mapping rate (%)	93.39	93.13

Supplementary Table 5. PCR primers and results for validation of contigs.

Primer name	Primer sequences (5′-3′)	Amplification	Sequencing	Identity
C1-F	ACGGACGGAGGAAGAGAAGGG	Failed		
C1-R	AAGGTGGTGGGCGAATGGAAT			
C2-F	AGAGAGGTGACCAATGGATGC	Yes	Yes	98%
C2-R	CGCTGTGTTACTGAGGACGAA			
C3-F	AATGTGGCTTGATGGTTAC	Yes	Yes	100%
C3-R	ATGATGGTGGTGTTTTTGA			
C4-F	CCCCAAAGAGAGTCCAATCG	Yes	Yes	96%
C4-R	GGCTCATCAGGGCTAACAAA			
C5-F	GGTGAAGGTTGGTTTGCGA	Yes	Yes	99%
C5-R	GACGAGGAGGGTGTGTGCT			
C6-F	TTTTTCTGGTGTGTTCGGTA	Yes	Yes	97%
C6-R	TCGTTCATTTGTGTTGGTTC			
C7-F	GACAGGAAAAAGAGAAGAAC	Yes	Yes	99%
C7-R	GAGGACAATAACGGAGAAAG			
C8-F	ATCCTATGCCCATTCACCCT	Yes	Yes	99%
C8-R	GCAGCTCCCAATTCTTTCGT			
C9-F	GAAGGCTGGGTCAGTGTTTA	Yes	Yes	96%
C9-R	GAGTGAGTTGGGAGTGTGTG			
C10-F	CTATGCGTTGGCTGTCTGT	Yes	Yes	95%
C10-R	TTTTGGGCGATTTTAATCT			
C11-F	ATGGGCTGAAGGGAAAATGA	Yes	Yes	98%
C11-R	AAGGAGCGGGTAGCGAACTG			
C12-F	TAAAAGGTTCACGAGTAGGG	Yes	Yes	99%
C12-R	TTGTGGTAGGTAACAGGCAG			
C13-F	GAGCCCGTCTGTTTTATCCTG	Yes	Yes	93%
C13-R	TACGCCTCTACCACTTCCCTG			
C14-F	CCACATCCATCCCTCACATCA	Yes	Yes	99%
C14-R	CACCTATCCTCACCGCCCTAT			
C15-F	GGAGGTGAGGTGCGATTGC	Yes	Yes	99%
C15-R	TCGTCGGCTGCTGGGTGTC			
C16-F	AAGAGTCAGAGGGGGGATTA	Yes	Yes	99%
C16-R	GTGGTAGAACTATGCGTGGC			
C17-F	CACTGCTTACCTACTTGCCT	Yes	Yes	98%
C17-R	CGTGTTGCTGTATATTGACG			
C18-F	AGGGTCAAAGAGGGTATGGA	Yes	Yes	96%
C18-R	TGAGCGGTATTTAGCGGAGA			

Supplementary Table 6. Evaluation of gene coverage using complete CDS sequences in GenBank and assembled transcriptomic datasets by Illumina sequencing.

Dataset		Number	Total length (bp)	Covered by assembly (%)	with >50% sequence in one scaffold	
					Number	Percentage
CDS	All	274	479,268	90.60	250	91.24
	>200bp	273	479,091	90.64	250	91.58
	>500bp	272	478,634	90.65	249	91.54
	>1000bp	193	419,270	90.95	176	91.19
Transcriptomes	>0bp	71,490	49,722,571	95.42	65,167	91.16
	>200bp	71,490	49,722,571	95.42	65,167	91.16
	>500bp	24,704	35,856,995	98.19	22,783	92.22
	>1000bp	12,249	27,252,964	99.09	11,249	91.84

Supplementary Table 7. BUSCO-based assessment of *E. sinensis* genome assembly based on arthropod and eukaryotic single-copy orthologs.

Gene set	BUSCO assessment results
Arthropod	C:85.9% [S: 82.6%, D: 3.3%], F: 4.2%, M: 9.9%, N:1066
Eukaryote	C:84.9% [S: 76.6%, D: 8.3%], F: 6.3%, M: 8.8%, N:303

Note: C, S, D, F and M stand for complete, complete and single-copied, complete but duplicated, fragmented, and missing orthologs in the assembled genome, respectively. N represents the total number of BUSCO arthropod and eukaryote (v. 2) genes.

Supplementary Table 8. Comparison of gene characteristics in *E. sinensis* and eight other arthropods.

Species	Number	Average transcript length (bp)	Average CDS length (bp)	Average exon number per gene	Average exon length (bp)	Average intron length (bp)
Eriocheir sinensis	28,033	5,017	1,078	3.26	330	1,602
Litopenaeus vannamei	25,596	8,889	1,546	5.94	260	1,484
Parhyale hawaiensis	30,603	17,955	2,041	3.64	220	6,068
Daphnia pulex	30,907	2,009	976	4.62	211	285
Drosophila melanogaster	13,689	4,261	1,621	3.97	408	888
Anopheles gambiae	14,324	5,955	1,583	4.21	376	1,363
Apis mellifera	11,062	8,976	1,627	6.46	252	1,347
Bombyx mori	14,623	6,030	1,224	5.44	225	1,082
Tribolium castaneum	16,531	5,289	1,351	4.34	311	1,179
Locusta migratoria	17,307	54,341	1,160	5.77	201	11,159

Supplementary Table 9. Summary of functional gene annotation of *E. sinensis*.

		Number	Percentage (%)
Total		28,033	100
Annotated	Nr	25,496	90.95
	Nt	23,870	85.15
	Swissprot	21,523	76.78
	COG	16,106	57.45
	KOG	20,361	72.63
	KEGG	19,667	70.16
	TrEMBL	21,845	77.93
	Iprscan	17,923	63.94
	GO	8,965	31.98
	Total annotated	26,117	93.17
Unannotated		1,916	6.83

Supplementary Table 10. Statistics of repetitive elements detected in the genomes of *E. sinensis* and five other crustaceans.

	Type	*Eriocheir sinensis*		*Litopenaeus vannamei*		*Parhyale hawaiensis*		*Hyalella azteca*		*Daphnia pulex*		*Eulimnadia texana*	
		Length (bp)	% in genome	Length (bp)	% in genome	Length (bp)	% in genome	Length (bp)	% in genome	Length (bp)	% in genome	Length (bp)	% in genome
Tandem repeat	Microsatellite	108,114,705	6.92	398,261,610	23.98	10,706,649	0.27	17,653,639	0.32	1,180,769	0.60	2,044,770	1.69
	Total	266,181,071	17.04	592,547,665	35.62	111,740,425	2.78	49,009,981	8.90	3,880,678	1.97	16,583,425	13.76
Interpersed repeat	DNA	223,157,308	14.28	529,392,649	31.82	474,227,451	11.79	48,059,508	8.72	8,499,275	4.31	17,445,139	14.47
	LINE	345,082,295	22.09	326,660,747	19.64	585,423,893	14.55	52,148,630	9.47	3,861,524	1.96	6,357,572	5.27
	SINE	2,744,831	0.18	1,039,518	0.06	1,294,093	0.03	117,002	0.02	1,837,683	0.93	360,649	0.30
	LTR	247,027,252	15.81	154,329,881	9.28	516,122,825	12.83	42,696,907	7.75	28,964,893	14.69	9,063,538	7.52
	Other	26,769	0	198	0.00	28,117	0	23,419	0.00	9,784	0	3,996	0.00
	Unknown	7,733,151	0.49	44,024,941	2.65	186,011,998	4.62	20,543,007	3.73	12,196,544	6.18	11,099,565	9.21
	Total	566,691,563	36.27	744,011,150	44.72	1,501,490,581	37.32	141,588,200	25.70	50,986,612	25.85	35,815,882	29.71
Total		707,661,693	45.3	899,214,214	54.05	1,604,189,772	39.87	188,496,585	34.22	52,769,598	26.76	43,622,227	36.19

Supplementary Table 11. Statistics of microsatellites detected in *E. sinensis* genome.

Type	Intergenic		Intron		Exon		Total	
	Length (bp)	% in TRs	Length (bp)	% in TRs	Length (bp)	% in TRs	Length (bp)	% in TRs
Mononucleotide	603,854	0.23	434,463	0.16	229	0	1,038,546	0.39
Dinucleotide	38,477,719	14.46	30,108,057	11.31	3,623	0	68,589,399	25.77
Trinucleotide	12,635,167	4.75	11,137,892	4.18	31,149	0.01	23,804,208	8.94
Tetranucleotide	3,472,623	1.3	3,513,615	1.32	1,052	0	6,987,290	2.63
Pentanucleotide	2,321,092	0.87	2,708,158	1.02	845	0	5,030,095	1.89
Hexanucleotide	1,375,166	0.52	1,284,521	0.48	5,480	0	2,665,167	1

TRs: tandem repeats.

Supplementary Table 12. GC-content distribution in the genomes of *E. sinensis* and other representative species.

Species	Total number of 200-bp windows	GC < 20%		20% ≤ GC ≤ 60%		GC > 60%	
		Number	Percentage (%)	Number	Percentage (%)	Number	Percentage (%)
E. sinensis	6,354,484	285,165	4.49	5,771,617	90.83	297,702	4.68
Portunus trituberculatus	5,676,837	134,906	2.38	5,390,585	94.96	151,346	2.67
Litopenaeus vannamei	8,042,542	1,090,770	13.56	6,590,652	81.95	361,120	4.49
Procambarus virginalis	6,395,341	4,609	0.07	6,192,232	96.82	198,500	3.10
Armadillidium vulgare	8,615,545	2,020,754	23.45	6,589,913	76.49	4,878	0.06
Parhyale hawaiensis	13,325,008	3,317	0.02	13,274,754	99.62	46,937	0.35
Hyalella azteca	2,708,485	8,081	0.30	2,659,158	98.18	41,246	1.52
Eurytemora affinis	1,941,708	26,528	1.37	1,914,073	98.58	1,107	0.06
Tigriopus californicus	940,270	256	0.03	933,879	99.32	6,135	0.65
Daphnia pulex	775,832	715	0.09	766,401	98.78	8,716	1.12
Eulimnadia texana	602,616	17,358	2.88	579,528	96.17	5,730	0.95
Drosophila melanogaster	711,377	2,408	0.34	693,193	97.44	15,776	2.22
Bombyx mori	2,092,249	10,480	0.50	2,047,947	97.88	33,822	1.62
Tribolium castaneum	756,146	11,170	1.48	741,154	98.02	3,822	0.51
Tetranychus urticae	446,365	474	0.11	445,872	99.89	19	0.00
Limulus polyphemus	8,216,301	31,920	0.39	8,184,104	99.61	277	0.00
Crassostrea virginica	3,422,691	6,339	0.19	3,412,743	99.71	3,609	0.11
Strigamia maritima	851,621	13,415	1.58	835,527	98.11	2,679	0.31
Patinopecten yessoensis	4,444,945	6,105	0.14	4,434,479	99.76	4,361	0.10
Apostichopus japonicus	3,991,296	22,086	0.55	3,963,358	99.3	5,852	0.15
Branchiostoma floridae	2,356,939	477	0.02	2,336,830	99.15	19,632	0.83
Homo sapiens	15,461,086	34,496	0.22	14,962,344	96.77	464,246	3.00

Supplementary Table 13. Overview of sample information and re-sequencing statistics.

Species	Sample ID	Geographic location	Gender	Raw reads data (bp)	Raw depth	Clean reads data (bp)	Map ratio	Effective depth
E. sinensis	Escm-1	Shanghai, China	Male	60,703,815,600	38.86	58,454,262,523	95.34%	37.42
E. sinensis	Escm-2	Shanghai, China	Male	56,371,941,900	36.08	54,403,377,577	95.07%	34.82
E. sinensis	Escm-3	Shanghai, China	Male	60,680,140,800	38.84	58,530,491,153	95.40%	37.47
E. sinensis	Escm-4	Shanghai, China	Male	54,509,661,600	34.89	52,672,248,245	95.29%	33.72
E. sinensis	Escm-5	Shanghai, China	Male	72,199,771,500	46.22	72,199,771,500	95.62%	46.22
E. sinensis	Eshf-1	Dongying, China	Female	61,685,059,500	39.48	59,107,774,877	94.94%	37.83
E. sinensis	Eshf-2	Dongying, China	Female	54,621,108,300	34.96	52,271,677,123	95.30%	33.46
E. sinensis	Eshf-3	Dongying, China	Female	55,246,968,900	35.36	52,661,733,312	95.28%	33.71
E. sinensis	Eshf-4	Dongying, China	Female	51,426,074,400	32.92	49,194,310,555	95.35%	31.49
E. sinensis	Eshf-5	Dongying, China	Female	67,113,468,300	42.96	63,692,372,346	95.58%	40.77
E. sinensis	Espf-1	Panjin, China	Female	54,197,604,600	34.69	51,887,322,562	94.99%	33.21
E. sinensis	Espf-2	Panjin, China	Female	67,617,211,800	43.28	64,148,397,114	95.18%	41.06
E. sinensis	Espf-3	Panjin, China	Female	58,635,499,800	37.53	55,773,840,807	94.93%	35.70
E. sinensis	Espf-4	Panjin, China	Female	56,281,331,100	36.03	51,819,671,041	94.37%	33.17
E. sinensis	Espf-5	Panjin, China	Female	66,907,331,400	42.83	63,043,495,651	93.49%	40.35

Supplementary Table 14. Distribution of SNPs within various genomic regions of mitten crabs.

Parameter	*E. sinensis*
Sample size	15
SNP sites	43,416,862
SNP Intergenic	38,189,678
SNP exon	498,682
Synonymous	317,138
nonSynonymous	176,144
nonSyn/Syn	0.55542
Stop gain	4,455
Stop loss	945
SNP Intron	3,624,510
SNP splicing	2,171
SNP downstream	517,723
SNP upstream	566,695
SNP upstream;downstream	17,403

Supplementary Table 15. Polymorphism analysis in ten resequenced *E. sinensis* individuals.

Sample ID		Total	Rate (%)	Intergenic	Rate (%)	Genic region	Rate (%)	Exon	Rate (%)	Intron	Rate (%)
Escm-1	SNP	12,639,546	1.200	11,208,819	1.189	1,009,395	0.900	121,473	0.675	887,922	0.943
	INDEL	3,205,401	0.304	2,885,143	0.306	233,075	0.208	12,011	0.067	221,064	0.235
Escm-2	SNP	12,594,775	1.194	11,156,290	1.182	1,004,819	0.892	131,540	0.711	873,279	0.927
	INDEL	3,163,678	0.300	2,847,554	0.302	230,259	0.204	11,843	0.064	218,416	0.232
Escm-3	SNP	12,594,843	1.194	11,165,347	1.183	1,011,735	0.903	118,833	0.664	892,902	0.948
	INDEL	3,174,102	0.301	2,854,708	0.303	232,627	0.208	11,848	0.066	220,779	0.234
Escm-4	SNP	12,544,374	1.193	11,110,952	1.182	1,003,041	0.892	129,740	0.705	873,301	0.929
	INDEL	3,109,950	0.296	2,797,209	0.298	227,622	0.202	11,710	0.064	215,912	0.230
Escm-5	SNP	12,487,097	1.184	11,049,286	1.171	1,003,592	0.892	132,204	0.717	871,388	0.926
	INDEL	3,203,660	0.304	2,880,726	0.305	234,748	0.209	12,172	0.066	222,576	0.237
Eshf-1	SNP	12,534,716	1.188	11,121,095	1.178	1,002,339	0.897	115,987	0.659	886,352	0.941
	INDEL	3,152,261	0.299	2,836,672	0.300	229,661	0.205	11,775	0.067	217,886	0.231
Eshf-2	SNP	12,530,614	1.191	11,110,352	1.181	1,007,469	0.901	115,676	0.655	891,793	0.948
	INDEL	3,126,451	0.297	2,812,644	0.299	228,527	0.204	11,666	0.066	216,861	0.230
Eshf-3	SNP	12,500,845	1.188	11,084,453	1.177	1,004,706	0.899	115,942	0.656	888,764	0.944
	INDEL	3,125,898	0.297	2,811,012	0.299	229,291	0.205	11,824	0.067	217,467	0.231
Eshf-4	SNP	12,505,633	1.192	11,087,385	1.182	1,007,306	0.902	115,355	0.652	891,951	0.949
	INDEL	3,103,003	0.296	2,791,277	0.298	227,123	0.203	11,689	0.066	215,434	0.229
Espf-1	SNP	12,138,836	1.155	10,778,319	1.146	963,922	0.863	111,209	0.631	852,713	0.907
	INDEL	3,115,770	0.296	2,802,858	0.298	227,530	0.204	11,823	0.067	215,707	0.229

Supplementary Table 16. Calibrations used in dating analysis (see Fig. 2a).

Taxon A	Taxon B	Time (MYA)	References
Eriocheir	*Portunus*	139-238	[3]
Tigriopus	*Eurytemora*	194-364	
Daphnia	*Eulimnadia*	128-298	
Tetranychus	*Litopenaeus*	568-642	
Daphnia	*Drosophila*	510-543	[4,5]
Tribolium	*Drosophila*	307-414	[4,6]

Supplementary Table 17. Gene copy numbers of representative expanded gene families of *E. sinensis*.

	F-H-ATPase	ABC family	thioredoxin	Hsp70	MnSOD	Thioredoxin peroxidase
DPULE	17	5	5	2	1	1
ETEX	26	5	6	2	1	1
EAFFI	30	20	19	3	1	1
TCALI	12	12	7	1	1	3
HAZTE	25	3	6	11	2	0
PHAW	15	4	0	5	0	1
LVANN	20	15	9	14	2	1
PTRIT	16	4	8	4	1	1
PVIRG	15	5	4	6	2	3
AVULG	19	8	11	11	2	0
BMORI	21	6	9	1	1	1
DMEAN	16	7	11	3	1	1
TCAST	12	13	7	2	1	1
TURTI	11	31	8	3	1	0
ESINE	102	37	28	20	19	19

Supplementary Table 18. Summary of genes located on Hox scaffolds in the *E. sinensis* genome.

Scaffold ID	GeneID	Location start	Location end	Stand
Scaffold_Hox_1	gene28	5214	6390	+
Scaffold_Hox_1	gene29	6580	10815	+
Scaffold_Hox_1	gene36	52782	53983	+
Scaffold_Hox_1	lab	63470	105071	-
Scaffold_Hox_1	pb	162835	166641	-
Scaffold_Hox_1	Hox3	393743	402812	-
Scaffold_Hox_1	gene76	442486	452337	+
Scaffold_Hox_1	miR-993	536334	536422	+
Scaffold_Hox_1	gene91	598288	602910	+
Scaffold_Hox_2	gene93	19538	20044	-
Scaffold_Hox_2	Dfd	329386	363583	-
Scaffold_Hox_2	miR-10	403728	403810	-
Scaffold_Hox_2	Scr	531071	608012	-
Scaffold_Hox_2	ftz	648227	653276	-
Scaffold_Hox_2	Antp	829249	869738	-
Scaffold_Hox_3	Ubx	154613	634114	-
Scaffold_Hox_3	abd-A	941655	1094885	-
Scaffold_Hox_3	miR-iab-4/8a	1389196	1389270	+
Scaffold_Hox_3	miR-iab-4/8b	1400542	1400616	+
Scaffold_Hox_3	gene317	1418564	1419299	+
Scaffold_Hox_4	Abd-B	111738	160053	-

Supplementary Table 19. The up-regulated IAG pathway genes in AG of *E. sinensis* after eyestalk ablation based on the KEGG analysis. EAT(C) A: EA means eyestalk ablation; T(C) means treatment group or control group; A means androgenic gland.

Name	EAT_A readcount	EAC_A readcount	Padj	KO ID	Description
SSR3	1983.2	973.99	0.034714	K13251	translocon-associated protein subunit gamma
SWP1, RPN2	4112.7	2015.41	0.036742	K12667	oligosaccharyltransferase complex subunit delta (ribophorin II)
HUGT	2855.07	1346.36	0.047246	K11718	UDP-glucose:glycoprotein glucosyltransferase
DNAJB11	572.09	245.29	0.02402	K09517	DnaJ homolog subfamily B member 11
CALR	16701.5	6980.85	0.037585	K08057	calreticulin
ADCY2	8.18	1.07	0.028149	K08042	adenylate cyclase 2
PRKG	31.53	10.79	0.044336	K07376	protein kinase, cGMP-dependent
STT3	4141.15	1833.52	0.027893	K07151	dolichyl-diphosphooligosaccharide--protein glycosyltransferase
GLRA2	20.9	6.44	0.03688	K05194	glycine receptor alpha-2

Supplementary Table 20. Primer sequences used in the Hox gene analysis.

Name	Sequence (5′-3′)
Ubx-RTF	CGCCAAGGACCAGAATG
Ubx-RTR	GGGTATCCCCGAGCAAC
AbdA-RTF	GCAATGCCGCTACTCCCA
AbdA-RTR	CGTGTATGTCTGTCTCCCTC
AbdB-RTF	GTGGTGTAACTATCCTCCGTA
AbdB-RTR	CTCAGGTAGCCGTCGTG
β-actin-RTF	GCATCCACGAGACCACTTACA
β-actin-RTR	CTCCTGCTTGCTGATCCACATC
miR-4-5p-RT	GGACGTATACTGAATGTATCCTGA
miR-8-3p-RT	GCACATTCAGTATACGTCCAA
Abd-A-mRNA probe	ACAGCAGCCGTCAACAGCCAAT
GFP-mRNA probe	GAGTTCAAGTCCATCTACATGG

Supplementary Table 21. Primer sequences used in the AG network analysis.

Name	Sequence (5′-3′)
NGL1_RTF	CCAAGAGGCAGTCATAGAT
NGL_RTR	ATGCCGTTGACTTTAGGTG
NEFH_RTF	ACCAAGGTCACGGTTTCCA
NEFH_RTR	GTCCGTCCTTCACGATGGT
SYT1_RTF	GAAAACCCACAGTTCAACG
SYT1_RTR	AGCCAAAAAGTATTCCCAA
DOP2R-2_RTF	TAGCGGCGTTTCTGGTGTG
DOP2R-2_RTR	TCGTGTAGATGACGGGGTT
FAS1-1_RTF	GGTTCCTTGTCTCCCGTTT
FAS1-1_RTR	GCTTGTGGTGCCGCTTTTT
NGEF-2_RTF	GGACACGGCTGACAAGTTC
NGEF-2_RTR	ATCTGACTCCATCGGGCAT
IAG1_RTF	GCCTTCCGTCCTTCTGCTA
IAG1_RTR	GCAGCCGTCGAGATGTTAG
IAG2_RTF	TTTTCAAGTCGTTAGGTCATCATTA
IAG2_RTR	CTTGATATGAGAGCTGAAGTCGTGG
SM_F1	CTATGCTTAATACATCGACTCAGCTTG
SM_R1	GTAAATAGTATGTGTTTGTATCCAATTCTTT
SM_F2	ATGTATTACAGAAATGCACCAG
SM_R2	TGTTCACCGATAAACCCTA
IAG_RTF	TCCGTCCTTCTGCTAATGCTG
IAG_RTR	CAGTGGGAGTGATTGGGAACA
iDMY _RTF	CACCCATCTACACCAAAAGC
iDMY _RTR	CTGCCCCACACAACCTCTCT
dsiDMY_F	TAATACGACTCACTATAGGGGCAACTGTGCCTTCTGTGA
dsiDMY_R	TAATACGACTCACTATAGGGGCTCTTCGTCCCTTCTTTT
dsEGFP-F	TAATACGACTCACTATAGGGCACAAGTTCAGCGTGTCCG
dsEGFP-R	TAATACGACTCACTATAGGGTGGGTGCTCAGGTAGTGGTT

Supplementary References

1. Tang, B.P. *et al.* High-quality genome assembly of *Eriocheir japonica sinensis* reveals its unique genome evolution. *Frontiers in Genetics* **10**(2020).
2. Song, L. *et al.* Draft genome of the Chinese mitten crab, *Eriocheir sinensis*. *GigaScience* **5**, 5 (2016).
3. Kumar, S., Stecher, G., Suleski, M. & Hedges, S.B. TimeTree: A Resource for Timelines, Timetrees, and Divergence Times. *Molecular Biology and Evolution* **34**, 1812-1819 (2017).
4. Benton, M.J., Donoghue, P.C.J. & Asher, R.J. *Calibrating and constraining molecular clocks*, (Oxford Univ. Press, 2009).
5. De Bie, T., Cristianini, N., Demuth, J.P. & Hahn, M.W. CAFE: a computational tool for the study of gene family evolution. *Bioinformatics* **22**, 1269-71 (2006).
6. Hahn, M.W., De Bie, T., Stajich, J.E., Nguyen, C. & Cristianini, N. Estimating the tempo and mode of gene family evolution from comparative genomic data. *Genome Research* **15**, 1153-60 (2005).
