# Supplementary material for: The Chinese mitten crab genome provides insights into adaptive plasticity and developmental regulation
Source: Nat Commun. 2021 Apr 22;12:2395. doi: 10.1038/s41467-021-22604-3 (PMC8062507; doi:10.1038/s41467-021-22604-3)
Supplement: Supplementary file 3 — Descriptions of Additional Supplementary Files [file 41467_2021_22604_MOESM3_ESM.docx]

Descriptions of Additional Supplementary Data

**Supplementary Data 1**

**Description:** List of 16 single-copy orthologous genes used in the phylogenetic analysis.

**Supplementary Data 2**

**Description:** GO enrichment analysis of shared gene families among four decapods.

**Supplementary Data 3**

**Description:** GO enrichment of E. sinensis-specific gene families.

**Supplementary Data 4**

**Description:** KEGG pathway enrichment of E. sinensis-specific gene families.

**Supplementary Data 5**

**Description**: GO enrichment analysis of the gene families expanded in the E. sinensis genome.

**Supplementary Data 6**

**Description:** KEGG pathway enrichment of the gene families expanded in the E. sinensis genome.

**Supplementary Data 7**

**Description:** Positive selected genes related to osmoregulation.

**Supplementary Data 8**

**Description:** PCR primers of osmoregulation-related genes used in this study.

**Supplementary Data 9**

**Description:** Anchored Hox gene-containing scaffolds on the reference genome (Hi-C linked).

**Supplementary Data 10**

**Description:** The expression of cofactor and downstream genes of Hox genes in the abdomen of E. sinensis from LM to J1 stage.

**Supplementary Data 11**

**Description:** The differentially expressed genes in the abdomen of E. sinensis from LM to J1 stage.

**Supplementary Data 12**

**Description:** Overrepresentation analysis of differentially expressed genes related to AG in the modules.

**Supplementary Data 13**

**Description:** Summary of gene coexpression network in the E. sinensis AG-related module (midnightblue).

**Supplementary Data 14**

**Description:** The annotation and differential expression of neuron-related genes in AG transcriptome of E. sinensis.

**Supplementary Data 15**

**Description:** Sequence IDs of members of the insulin-relaxin superfamily used to construct the phylogenetic depicted in Supplementary Fig. 33.

**Supplementary Data 16**

**Description:** KEGG enrichment of the target genes of differently expressed miRNA in the androgenic gland between synthesis and secretion phase.

**Supplementary Data 17**

**Description:** The differentially expressed proteins involved in IAG secretion.

**Supplementary Data 18**

**Description:** The expression of potential genes for IAG pathway in AG of E. sinensis after eyestalk ablation.
