# Supplementary material for: The Chinese mitten crab genome provides insights into adaptive plasticity and developmental regulation
Source: Nat Commun. 2021 Apr 22;12:2395. doi: 10.1038/s41467-021-22604-3 (PMC8062507; doi:10.1038/s41467-021-22604-3)
Supplement: Supplementary file 22 — Reporting Summary [file 41467_2021_22604_MOESM22_ESM.pdf]

nature research

Corresponding author(s): Zhaoxia Cui, Fuhua Li and Ka Hou Chu

Last updated by author(s): Feb 12, 2021

Reporting Summary

Nature Research wishes to improve the reproducibility of the work that we publish. This form provides structure for consistency and transparency in reporting. For further information on Nature Research policies, see our Editorial Policies and the Editorial Policy Checklist.

Statistics

For all statistical analyses, confirm that the following items are present in the figure legend, table legend, main text, or Methods section.

n/a	Confirmed	
☐	☒	The exact sample size (*n*) for each experimental group/condition, given as a discrete number and unit of measurement
☐	☒	A statement on whether measurements were taken from distinct samples or whether the same sample was measured repeatedly
☐	☒	The statistical test(s) used AND whether they are one- or two-sided<br>*Only common tests should be described solely by name; describe more complex techniques in the Methods section.*
☐	☒	A description of all covariates tested
☐	☒	A description of any assumptions or corrections, such as tests of normality and adjustment for multiple comparisons
☐	☒	A full description of the statistical parameters including central tendency (e.g. means) or other basic estimates (e.g. regression coefficient) AND variation (e.g. standard deviation) or associated estimates of uncertainty (e.g. confidence intervals)
☐	☒	For null hypothesis testing, the test statistic (e.g. *F*, *t*, *r*) with confidence intervals, effect sizes, degrees of freedom and *P* value noted<br>*Give P values as exact values whenever suitable.*
☐	☒	For Bayesian analysis, information on the choice of priors and Markov chain Monte Carlo settings
☒	☐	For hierarchical and complex designs, identification of the appropriate level for tests and full reporting of outcomes
☒	☐	Estimates of effect sizes (e.g. Cohen's *d*, Pearson's *r*), indicating how they were calculated

Our web collection on statistics for biologists contains articles on many of the points above.

Software and code

Policy information about availability of computer code

Data collection	Sequencing data were obtained using software available with sequencing machines: HiSeq 2000, HiSeq 2500, HiSeq4000 and HiSeq X Ten, Pacific Biosciences RS II and BGISEQ-500.
Data analysis	DISCOVAR de novo v52488; Falcon v1.7.4; HABOT2; SOAPdenovo2 v2.04-r241; BLAT v36; bowtie2 v2.2.5; mafft v6.864b; muscle v3.7; bwa v0.7.12-r1039; lastz v1.03.73; blastall v2.2.21; hcluster_sg v0.5.1; ALLMAPS v0.6.9; HiC-Pro v2.8.0_devel; juicer v1.5; 3D-DNA v170123; RepeatMasker v4.0.6; RepeatProteinMask v4.0.6; RepeatModeller v1.0.8; Tandem Repeats Finder v4.0.7; GeneWise v2.4.1; Augustus v3.0.2; GENSCAN v1.0; GlimmerHMM v3.0.4; SNAP v2006-07-28; GLEAN v1.0.1; Cufflinks v2.2.1; OrthoMCL v1.02; PhyML v3.0; PAML v4.4; CAFE v1.6; MEGA5; ClustalW v2.1; SMART v5.0; SPSS 16.0; SAMtools v1.2; EIGENSOFT v7.2.1; STRUCTURE v.2.3.4; the pairwise sequentially Markovian coalescence (PSMC); Haploview v4.2; VCFtools v0.1.13; TBLASTN v2.2.26; edgeR v3.22.5; BD FACS Diva v3.0.

For manuscripts utilizing custom algorithms or software that are central to the research but not yet described in published literature, software must be made available to editors and reviewers. We strongly encourage code deposition in a community repository (e.g. GitHub). See the Nature Research guidelines for submitting code & software for further information.

Data

Policy information about availability of data

All manuscripts must include a data availability statement. This statement should provide the following information, where applicable:

- Accession codes, unique identifiers, or web links for publicly available datasets
- A list of figures that have associated raw data
- A description of any restrictions on data availability

The Eriocheir sinensis genome data have been deposited at NCBI under the accession code CL100111224_L02 (https://www.ncbi.nlm.nih.gov/sra/?term=CL100111224_L02). 10X Genomics data were deposited at the NCBI under the BioProject number PRJNA238496 (https://www.ncbi.nlm.nih.gov/bioproject/?

term=PRJNA238496). The genomic Hi-C sequencing data were deposited in the Sequence Read Archive (SRA) database at SRR10802271 (https://www.ncbi.nlm.nih.gov/sra/?term=SRR10802271). RNA-Seq data used for annotation and biological analyses include the following: NCBI SRA SRR2180019-SRR2180020 (https://trace.ncbi.nlm.nih.gov/Traces/sra/?study=SRP062750), SRR770582 (https://www.ncbi.nlm.nih.gov/sra/?term=SRR770582), SRR769751 (https://www.ncbi.nlm.nih.gov/sra/?term=SRR769751), SRR1199039 (https://www.ncbi.nlm.nih.gov/sra/?term=SRR1199039), SRR1199058 (https://www.ncbi.nlm.nih.gov/sra/?term=SRR1199058), SRR1205971 (https://www.ncbi.nlm.nih.gov/sra/?term=SRR1205971), SRR1199228 (https://www.ncbi.nlm.nih.gov/sra/?term=SRR1199228), SRR2170964 (https://www.ncbi.nlm.nih.gov/sra/?term=SRR2170964), SRR2170970 (https://www.ncbi.nlm.nih.gov/sra/?term=SRR2170970), SRR10058623-SRR10058634 (https://trace.ncbi.nlm.nih.gov/Traces/sra/?study=SRP220350), SRR10083958-SRR10083963 (https://trace.ncbi.nlm.nih.gov/Traces/sra/?study=SRP220979), SRR10276365-SRR10276369 (https://trace.ncbi.nlm.nih.gov/Traces/sra/?study=SRP225577), SRR10276537-SRR10276548 (https://trace.ncbi.nlm.nih.gov/Traces/sra/?study=SRP225587), SRR13644341-SRR13644350 (https://www.ncbi.nlm.nih.gov/bioproject/?term=PRJNA699917), SRR13664056-SRR13664067 (https://www.ncbi.nlm.nih.gov/bioproject/?term=PRJNA700787) and PRJNA700687 (https://www.ncbi.nlm.nih.gov/bioproject/?term=PRJNA700687). The E. sinensis genome sequences are also be available at the genome website (http://www.genedatabase.cn/esi_genome.html).

Field-specific reporting

Please select the one below that is the best fit for your research. If you are not sure, read the appropriate sections before making your selection.

[x] Life sciences [ ] Behavioural & social sciences [ ] Ecological, evolutionary & environmental sciences

For a reference copy of the document with all sections, see nature.com/documents/nr-reporting-summary-flat.pdf

Life sciences study design

All studies must disclose on these points even when the disclosure is negative.

Sample size	Sample sizes were determined according to previous data published by us (Ref. 26) and others (Ref. 16). We sequenced the genome of a single male adult of Eriocheir sinensis. A total of 15 crab individuals were selected for genome resequencing. Four and three replicates were used for qPCR experiments presented in Figure 3d and Figure 4d, respectively.
Data exclusions	No data were excluded.
Replication	Experimental findings were reliably reproduced.
Randomization	The samples were randomly allocated in the experiments.
Blinding	No blinding was applied to this study as this technique does not apply to our work.

Reporting for specific materials, systems and methods

We require information from authors about some types of materials, experimental systems and methods used in many studies. Here, indicate whether each material, system or method listed is relevant to your study. If you are not sure if a list item applies to your research, read the appropriate section before selecting a response.

Materials & experimental systems

n/a	Involved in the study	
x		Antibodies
x		Eukaryotic cell lines
x		Palaeontology and archaeology
	x	Animals and other organisms
x		Human research participants
x		Clinical data
x		Dual use research of concern

Methods

n/a	Involved in the study	
x		ChIP-seq
	x	Flow cytometry
x		MRI-based neuroimaging

Animals and other organisms

Policy information about studies involving animals; ARRIVE guidelines recommended for reporting animal research

Laboratory animals	Species: Eriocheir sinensis, Strain: an inbred from six generations of small-size population mating or wild; Sex: Female or male; Age: embryos, larvae, adults
Wild animals	The mitten crabs used for whole-genome resequencing were 15 wild individuals of E. sinensis. The detailed sample information including geographic location and sex is in Supplementary Table 13. The samples were captured and then send to the lab for DNA extraction.
Field-collected samples	The male mitten crab E. sinensis used for genome sequencing is an inbred from six generations of small-size population mating produced by Panjin Guanghe Crab Industry Co., Ltd. This crab with body weight approximately 150 g was captured and then send to

	the lab for DNA extraction.
Ethics oversight	All animals were handled and treated according to the guidelines approved by the Animal Ethics Committee [2020(37)] at Institute of Oceanology, Chinese Academy of Sciences (Qingdao, Shandong, China).

Note that full information on the approval of the study protocol must also be provided in the manuscript.

Flow Cytometry

Plots

Confirm that:

- [x] The axis labels state the marker and fluorochrome used (e.g. CD4-FITC).
- [x] The axis scales are clearly visible. Include numbers along axes only for bottom left plot of group (a 'group' is an analysis of identical markers).
- [x] All plots are contour plots with outliers or pseudocolor plots.
- [x] A numerical value for number of cells or percentage (with statistics) is provided.

Methodology

Sample preparation	Appendages were collected from crab individuals, and mouse (genome size of 2.50 Gb) blood cells were used as internal standard. Briefly, samples of tissues were chopped with a razor blade in the buffer of PBS. 1 mL of the homogenized cell suspension was filtered through a 30 μm nylon filter, added with 12 μL of propidium iodide (50mg/mL), and stained with 2 μL of RNase (10 mg/mL) for 20 mins.
Instrument	BD FACSAria II
Software	BD FACSDiva3.0
Cell population abundance	No post-fractions were collected, and preliminary FSC/SSC gates for the starting cell population were not used.
Gating strategy	SSC×FSC to include crab hemocyte, PE-Texas Red-H×PE-Texas Red-W to include G0 stage cell

- [x] Tick this box to confirm that a figure exemplifying the gating strategy is provided in the Supplementary Information.
